# Dynamic Switch of Negative Feedback Regulation in *Drosophila* Akt–TOR Signaling

**DOI:** 10.1371/journal.pgen.1000990

**Published:** 2010-06-17

**Authors:** Lutz Kockel, Kimberly S. Kerr, Michael Melnick, Katja Brückner, Matthias Hebrok, Norbert Perrimon

**Affiliations:** 1 Department of Genetics and Howard Hughes Medical Institute, Harvard Medical School, Boston, Massachusetts, United States of America; 2 Diabetes Center, Department of Medicine, University of California San Francisco, San Francisco, California, United States of America; 3 Cell Signaling Technology, Beverley, Massachusetts, United States of America; 4 Department of Cell and Tissue Biology, University of California San Francisco, San Francisco, California, United States of America; Stanford University School of Medicine, United States of America

## Abstract

Akt represents a nodal point between the Insulin receptor and TOR signaling, and its activation by phosphorylation controls cell proliferation, cell size, and metabolism. The activity of Akt must be carefully balanced, as increased Akt signaling is frequently associated with cancer and as insufficient Akt signaling is linked to metabolic disease and diabetes mellitus. Using a genome-wide RNAi screen in *Drosophila* cells in culture, and *in vivo* analyses in the third instar wing imaginal disc, we studied the regulatory circuitries that define dAkt activation. We provide evidence that negative feedback regulation of dAkt occurs during normal *Drosophila* development *in vivo*. Whereas in cell culture dAkt is regulated by S6 Kinase (S6K)–dependent negative feedback, this feedback inhibition only plays a minor role *in vivo*. In contrast, dAkt activation under wild-type conditions is defined by feedback inhibition that depends on TOR Complex 1 (TORC1), but is S6K–independent. This feedback inhibition is switched from TORC1 to S6K only in the context of enhanced TORC1 activity, as triggered by mutations in *tsc2*. These results illustrate how the Akt–TOR pathway dynamically adapts the routing of negative feedback in response to the activity load of its signaling circuit *in vivo*.

## Author Summary

The development of multi-cellular organisms depends on the precise choreography of a diverse array of signal transduction pathways. This requires balanced regulation by activating as well as repressing signals. Negative feedback, defined as a signaling response counteracting the stimulus, is a frequently used mechanism to dampen signaling pathway activity. Accordingly, loss of negative feedback is often observed during progression of cancer, while constitutive engagement of negative feedback contributes to chronic loss-of-function phenotypes. Ectopic activation of the Akt–TOR pathway is frequently associated with tumor susceptibility and cancer and contributes to obesity-induced metabolic disease and type II diabetes. Using *Drosophila* cell culture and the developing fly, we dissect the regulatory circuitry defining negative feedback regulation of dAkt. Our work shows that dAkt activity is regulated by two qualitatively different negative feedback mechanisms and that the activity level of the dAkt pathway dictates which feedback mechanism is utilized. Under normal physiological activity conditions, we observe a feedback mechanism that is dependent on TOR complex 1, but independent of S6K. Under conditions of pathological high pathway activity, we observe an S6K–dependent negative feedback mechanism. Our identification of a quantitative-to-qualitative switch in dAkt–TOR negative feedback signaling might have important implications in the biology of cancer and metabolic diseases.

## Introduction

The development of multi-cellular organisms depends on the precise choreography of a diverse array of signal transduction pathways. Besides the requirement of some signaling events to occur in a spatial or temporal on-off manner, other pathways need to stay homeostatically active within physiological boundaries. This requires balanced regulation by activating as well as repressing signals.

Mechanistically, three basic concepts of downregulating signaling pathway have emerged: (1) control via specific inhibitory ligands or receptors [1], [2], (2) negative cross-regulation by distinct signaling pathways [3], and (3) auto-regulation by negative feedback mechanisms [4], [5]. In most cases, the molecular component that executes the feedback-mediated inhibition is transcriptionally targeted by the very pathway that it regulates. This mechanism ensures an interdependence of signaling activity and feedback regulation and is often viewed as an inherent means to downregulate signaling pathways after stimulation.

Loss of negative feedback regulation has been correlated with the initiation, growth and progression of tumors. For example, loss of negative feedback in Hedgehog (Hh) signaling by impeding *patched* function results in ectopic Hh signaling, basal cell carcinoma and medulloblastomas [6]. The expression of *axin2* or *dickkopf-1*, which encode feedback inhibitors of Wnt signaling, is silenced in colon and breast carcinomas and early lung adenocarcinoma [7], [8]. Negative feedback regulators of Ras signaling, such as Sprouty proteins and MAPK phosphatases, are downregulated in liver, prostate and breast cancers [9], [10], [11], [12]. Similarly, inhibition of negative feedback regulation has been reported for JAK/STAT, TGF-beta and NF-kappaB signaling pathways [13], [14], [15]. These observations indicate that some cancers arise by “breaching” auto-regulatory control mechanisms of signaling pathways via mutational inactivation or epigenetic silencing of negative feedback regulators.

The Akt-TOR pathway has emerged as a central signaling nexus that integrates responses to growth factors, nutrients, metabolites and stress. Most prominently, activation of Akt is initiated by the insulin receptor (InR), relayed via an intracellular signaling cascade comprising insulin receptor substrate (IRS), class IA PI3 Kinase (PI3K), PDK1 and TOR complex 2 (TORC2), consisting of TOR, Rictor, Sin1, Lst8 and PRR5L [16], [17], [18], [19], [20]. Among other substrates, Akt inhibits the activities of the transcription factor FoxO [21] and the Rheb-specific GTPase activating protein (GAP) Tsc2. In turn, Rheb regulates the TOR complex 1 (TORC1), containing TOR, Raptor and Lst8 [17], [22], [23]. TORC1 targets several well characterized substrates, most notably S6 Kinase (S6K) [24], [25]. Hence, the two distinct TOR complexes TORC1 and TORC2 both participate in Akt-TOR signaling, but act at different levels in the Akt-TOR signaling pathway and integrate distinct stimuli. TORC2 responds to growth factors and might determine the substrate specificity of Akt [26], [27], [28], [29], while TORC1 mediates signaling by amino acids and cellular energy stress [30], [31], [32], [33]. Ectopic activation of the core Akt-TOR signaling pathway by a variety of mechanisms is a frequent event in cancer biology 18,34. Moreover, chronic diseases such as obesity and type II diabetes show pathological alteration of Akt-TOR activity [35].

Negative feedback mechanisms regulate the signaling input into the Akt-TOR pathway. Indeed, FoxO transcription factors inhibit the activity of the phosphatases PP2A and calcineurin by driving the expression of Atrogin-1, causing elevated levels of Akt phosphorylation and activity [36], [37]. Furthermore, Akt-dependent inhibition of the FoxO transcription factor results in reduced transcription of the *inR* gene. Conversely, low Akt-TOR signaling selectively increases *InR* mRNA translation relative to the total mRNA pool. In conjunction, both mechanisms reduce the relative levels of InR expression when Akt-TOR activity is high, thereby desensitizing against a stimulating ligand [38], [39], [40]. In addition, an S6K-dependent negative feedback mechanism leads to IRS1 destabilization, thus decreasing Akt activity [41], [42], [43], [44]. While these negative feedback mechanisms have been defined in cell culture, it is currently unknown whether and how feedback regulation within the Akt-TOR signaling pathway is exerted during development *in vivo*.

In *Drosophila*, the dAkt-TOR signaling pathway is conserved and regulates cell proliferation, and developmental timing and sizing of cells, organs and the whole fly [45], [46], [47]. As with the mammalian counterparts, *Drosophila* Akt receives regulatory inputs from TORC2 as well as PDK1. The phosphorylation site in the C-terminal hydrophobic motif of *Drosophila* Akt is conserved, and, while dispensable for normal *Drosophila* development, is required for relaying high PI3K signaling levels [29], [48], [49], [50]. Similarly, prostate-specific ablation of C-terminal Akt phosphorylation in mice conditionally mutant for *Rictor* delays lethality of *Pten* ^+/-^ induced prostate cancer [51]. In general, the C-terminal phosphorylation of Akt has emerged as a valuable and reliable tool to detect Akt activity *in vivo* and *in vitro* [52], [53]. In contrast to the three *Akt* genes in mammalian genomes, *Drosophila* contains only a single *dAkt* gene. In addition, the InR and IRS families are represented solely as single genes, and the insulin/InR-related IGF-1/IGFR system is absent in flies. This simplicity underscores the suitability of the fly as a model organism for studying complex processes like the *in vivo* analysis of feedback mechanisms.

To date, the analysis of feedback-mediated Akt-TOR pathway adaptation has been pursued under genetic or metabolic conditions that trigger high, possibly supra-physiological activity of TORC1 and S6K, and mostly in cell culture systems [18], [54]. In this study, we present evidence that regulation by negative feedback is an integral part of the dAkt-TOR pathway *in vivo*. Importantly, we demonstrate that the pathway utilizes two distinct modes of negative feedback to downregulate its activity *in vivo*, independently of FoxO. Conditions of wild-type TORC1 activity favor a dampening feedback signal emanating from TORC1 itself, independent of S6K. In contrast, conditions that induce high TORC1 activity trigger an S6K-dependent feedback mechanism to dampen dAkt-TOR pathway signaling. Our observations suggest that S6K acts as a load-sensitive regulator of Akt-TOR signaling. We propose the presence of a novel dual “overload protection” circuit that emphasizes the importance of tight control over Akt-TOR pathway signal levels.

## Results

### An assay for *Drosophila* phospho-Akt

We established a cell-based assay for regulators of insulin signaling in *Drosophila* that could be used in a genome-wide RNAi screen. Testing of more than 64 commercially available phospho-antibodies against components of this signaling cascade revealed that none of them recognized an insulin-induced antigen using immunohistochemistry (data not shown). Thus, we generated a phospho-Akt antiserum recognizing the phosphorylation of the C-terminal hydrophobic motif of *Drosophila* Akt. The single *dakt* gene encodes two splice forms of 513 and 611 amino acids in length. The antibody (hereafter referred to as anti P-dAkt) recognizes two bands in a western blot assay, likely corresponding to the phosphorylated forms of the short and long splice form, respectively.

**Figure 1:**
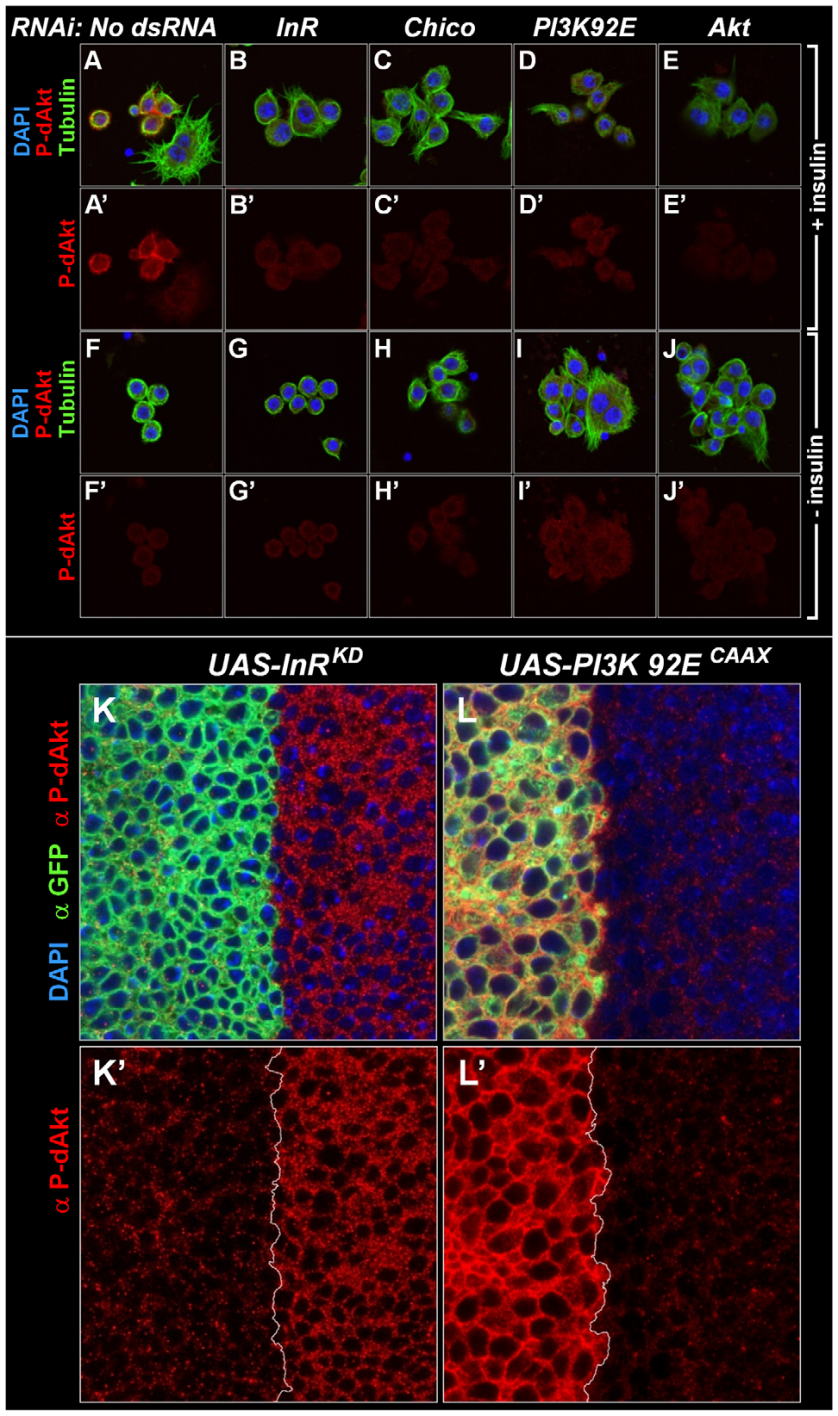
Specificity of anti P-dAkt. (A-J') *Drosophila* Kc_167_ cells stained with DAPI (blue), anti P-dAkt (red), and anti alpha-tubulin (green) after 10 minutes of insulin stimulation (A-E') or at baseline without insulin stimulus (F-J'). Cells were RNAi treated as described in the experimental procedures using no dsRNA (A, A'), *InR* dsRNA (B, B', G, G'), *Chico* dsRNA (the IRS ortholog, C, C', H, H'), *PI3K93E* dsRNA (the catalytic subunit of the class IA PI3-Kinase, D, D', I, I') and *dAkt* (E, E', J, J'). Note that large polynucleated cells are resistant to the insulin stimulus (A, A'). (K-L') Single tangential optical sections of third instar wing imaginal discs. The region of the dorso-ventral (D/V) boundary at the future wing pouch is shown, dorsal to the left. Wing discs are stained with DAPI (blue), anti P-dAkt (red) and anti-GFP (green), marking the expression domain of *apterous-Gal4* and the *UAS-InR^DN^* (K, K') and *UAS-PI3K^CAAX^* (L, L') expression constructs. K' and L' show P-dAkt channels only, the D/V compartment boundary is marked by a white line.

In order to test if the phosphorylation of this hydrophobic motif correlates well with activity of Akt [52], [53], we stimulated *Drosophila* Kc_167_ cells with insulin for 10 min and induced a robust P-dAkt signal. The hydrophobic motif phosphorylation was strongly suppressed when known components of the insulin signaling cascade, including InR, Chico, the catalytic subunit of PI3K, PI3K92E and dAkt itself were silenced by RNAi (Figure 1A–1E and 1A'–1E'). We next asked whether the anti P-dAkt antibody detected differences in dAkt phosphorylation in the third instar imaginal disc, an established system to study cell and tissue size alterations dependent InR signaling *in vivo* [55], [56], [57]. To validate our assay, we expressed dominant negative insulin receptor (InR^DN^) or a constitutively active catalytic subunit of PI3K (PI3K^CAAX^), utilizing the UAS-Gal4 expression system [58]. Using apterous-Gal4 (ap-Gal4) to drive expression of InR^DN^ and PI3K^CAAX^ concomitant with membrane-tagged GFP in the dorsal compartment of the wing disc, we compared the levels of P-dAkt immunoreactivity in the dorsal compartment cells to non-expressing ventral cells as controls (Figure 1K–1L and 1K'–1L'). Expression of InR^DN^ resulted in a reduction of P-dAkt levels (Figure 1F and 1F'), whereas PI3K^CAAX^ expression drastically increased the P-dAkt intensity when compared to ventral control cells (Figure 1G and 1G'). Staining of wild-type imaginal wing discs did not reveal a pattern of P-dAkt immunoreactivity associated with compartments or their boundaries (not shown).

Western blotting of extracts of Kc_167_ cells treated with various dsRNAs against components of the insulin signaling pathway confirmed the specificity found by immunostaining of cells and *Drosophila* tissue (Figure S1). RNAi-mediated knockdown of InR, PI3K92E or dAkt abolished the anti-P-dAkt reactivity. Together, our data show that anti P-dAkt faithfully detects dAkt phosphorylation, and that the hydrophobic phosphorylation motif correlates with InR-PI3K regulated dAkt activity in cell culture and *in vivo*.

### A genome-wide RNAi screen for regulators of P-dAkt reveals negative feedback regulation by Tsc1/Tsc2-TOR-S6K signaling

To identify novel regulatory inputs in the insulin signal transduction pathway, we used the Cytoblotting/In Cell Western method in combination with the newly generated anti P-dAkt antibody (Figure S2A) as a fast and quantitative cell-based high throughput assay. Cells were grown in 384-well plates and, after three days in the presence of gene-specific dsRNAs of the genome-wide dsRNA library [59], were fixed and immunostained with anti P-dAkt antiserum. Bound primary antibody was quantified and normalized to cell number. Using this approach, we carried out genome-wide RNAi screens in duplicates without stimulation and after 10 min. of insulin stimulation. We identified 79 dsRNAs that conferred suppression of dAkt phosphorylation, and 56 dsRNAs that enhanced P-dAkt immunoreactivity (Table S1). Importantly, five out of eight known components functioning upstream of dAkt were identified, validating the reliability of this method (Figure S2B, S2C, and Table S1). dsRNAs against *Chico*, PHLLP and *Pten*, the remaining 3 regulators of dAkt, scored below the cutoff threshold.

**Figure 2:**
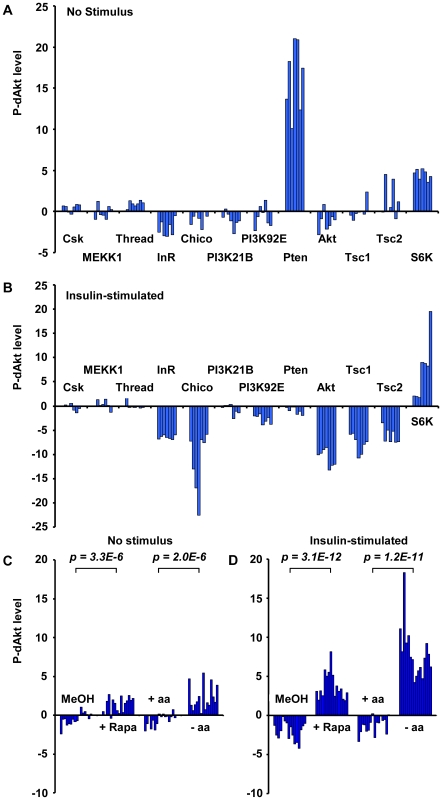
Phosphorylation of Akt is regulated by the activity of the InR-PI3K as well as the Tsc1/Tsc2-TOR signaling branch. (A, B) P-dAkt levels (expressed as calculated Z-Scores, defined as the difference from the average, expressed as multiples of the standard deviation) for independently synthesized dsRNAs against the InR-PI3K and Tsc1/Tsc2-TOR signaling pathway branches under baseline (A) or insulin-stimulated (B) conditions. (C, D) P-dAkt phosphorylation levels (expressed as calculated Z-Scores) at baseline (C) and after insulin stimulation (D) of Kc_167_ cells pretreated with 50 nM Rapamycin (Rapa) for 4 hrs or amino acid (-aa) and serum-free tissue culture medium (8 hrs). Methanol (MeOH) and aa-containing medium (+aa) were used as control, respectively. Experiments were analyzed using external standard curves as described in experimental procedures. *P-values* were calculated using the two-tailed students *t-test*.

In our screens, we found that dsRNAs against the small GTPase *Rheb*, the TORC1 component *Raptor* and *S6K*, all downstream mediators required for insulin signal transduction, induced enhanced phosphorylation of dAkt in the absence of insulin. Conversely, dsRNAs against the negative regulators *Tsc1* and *Tsc2* suppressed the P-dAkt signal when the pathway was activated by insulin. In total, we identified ten out of eleven components known to participate in the Tsc1/Tsc1-TOR signaling branch, with Tctp [60] as the single component not identified by any of our screens (Figure S2 and Table S1). Interestingly, the function of Tctp as a regulator of Rheb is controversial [61], [62]. The results of the genome-wide RNAi screen were validated using independent dsRNAs against known insulin pathway components (Figure 2A and 2B). dsRNAs against *CSK*, *MEKK1* and *Thread* were used as negative controls, and *Pten* dsRNA as positive control. As observed in the genome-wide screen, removal of the negative regulators Tsc1 and Tsc2 resulted in suppression of P-dAkt in the presence of insulin, while knock down of S6K elevated P-dAkt at baseline conditions. Thus, dAkt phosphorylation is sensitive to interference by Tsc1/Tsc2-TOR-S6K signaling, classically viewed as signaling downstream of dAkt [23], [63], [64], [65], [66], [67]. These results are consistent with the existence of an inhibitory feedback signal by the components downstream of dAkt, namely Rheb, Raptor, Tsc1/2 and S6K [50], [65].

To test the feedback by different means than RNAi, two different strategies were used to inhibit the activator of S6K, TORC1 (Figure 2C). In a chemical approach, we exposed cultured cells to rapamycin, an effective, small molecule inhibitor of TORC1 [68], [69], [70]. In a metabolic approach, we starved cultured cells in amino acid-free media, thereby potently inhibiting TORC1 activity [65], [68], [69], [70], [71]. Rapamycin-induced TORC1 inhibition and amino acid starvation both led to a highly significant increase in P-dAkt compared to control cells treated with solvent control or amino acid-containing medium, respectively. These results confirm the RNAi data and validate the existence of a negative feedback loop that regulates the activation of the pathway by insulin [65].

### Activation of S6K correlates with an inhibition of P-dAkt

**Figure 3:**
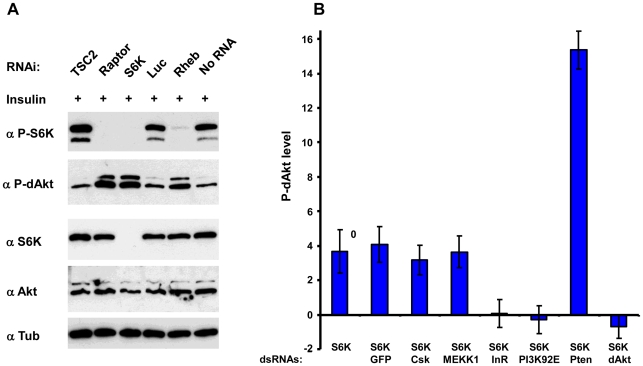
Inhibition of S6K results in derepression of Akt by inhibition of InR. (A) Immunoblots of total lysates prepared from *Drosophila* Kc_167_ cells after 10 min of insulin stimulation treated with dsRNAs against *Tsc2*, *Raptor*, *S6K*, *Luciferase* and *Rheb* as indicated. Non-RNAi treated and luciferase (luc) dsRNA treated cells were used as negative controls. (B) InR and PI3K are required to mediate S6K dependent negative feedback on dAkt. Calculated Z-scores of P-dAkt derived from a cytoblot/in cell western under non-stimulated (no insulin), S6K RNAi treated condition, using untreated cells as reference. DsRNAs are utilized as indicated, RNAi against *GFP*, *CSK* and *MEKK* are used as negative controls, RNAi against *Pten* and *Akt* as positive controls. Values and their SDs are calculated from seven replicates. Please note that dAkt phosphorylation is exclusively derived from removing S6K dependent negative feedback.

Since dsRNA-mediated knockdown of S6K enhanced dAkt phosphorylation, we asked whether the inhibitory effect of S6K on dAkt phosphorylation was related to its activity. The activation of *Drosophila* S6K can be scored using phosphorylation of Thr 398 (orthologous to Thr 389 in mammalian S6K1) as readout (Figure 3A) [50], [65]. We analyzed lysates of *Drosophila* Kc_167_ cells pretreated with dsRNAs against *Tsc2*, *Raptor*, *S6K* and *Rheb*, for both S6K and dAkt phosphorylation. Cells treated with dsRNA against luciferase and non-RNAi treated cells served as negative controls (Figure 3A, lanes 4 and 6). Enhanced P-dAkt reactivity correlated with suppression of S6K phosphorylation, with a clear elevation of dAkt phosphorylation when Rheb, Raptor or S6K expression was knocked down.

To address how S6K mediates its feedback inhibition of dAkt phosphorylation, we induced dAkt phosphorylation by exclusively removing the negative feedback inhibition in Kc_167_ cells using RNAi against S6K in the absence of insulin stimulation. The robust enhancement of P-dAkt due to the knockdown of S6K expression was not affected by further RNAi-mediated knockdown of control genes such as *GFP*, *CSK* or *MEKK1/4*. We then knocked down the individual components of the insulin signaling pathway to assess whether they were required for the enhanced dAkt phosphorylation caused by S6K silencing (Figure 3B). In the *S6K^RNAi^* background, RNAi-mediated silencing of *Pten* (positive control) further enhanced the P-dAkt levels, while dsRNA to *dAkt* (negative control) reduced P-dAkt to baseline levels. Importantly, RNAi against the signaling effectors *InR* or *PI3K* suppressed the enhanced dAkt phosphorylation conferred by S6K RNAi, indicating that the S6K-dependent feedback inhibition requires the functions of these two upstream signaling effectors.

### Phosphorylation of dAkt *in vivo* is regulated by negative feedback

**Figure 4:**
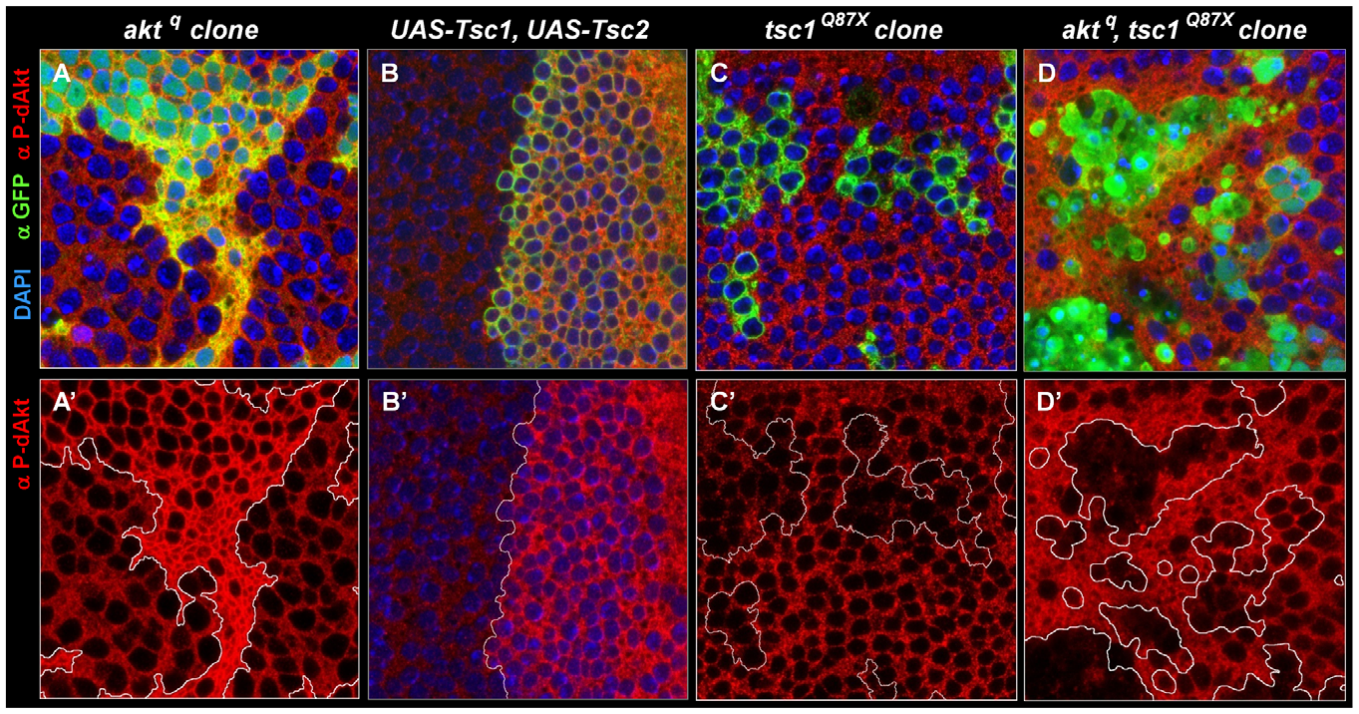
The Akt–TOR signal transduction pathway has a circular structure *in vivo*. Single tangential optical sections (A,A'-D,D') of third instar wing imaginal discs stained with DAPI (A-D, blue), anti P-dAkt (A-D', red) and anti-GFP (A-D, green). (A, A') MARCM, M^+^ clone of *akt^q^* located at the wing primordium. (B, B') View of the dorso-ventral boundary at the future wing pouch. GFP expression (green) marks the dorsal expression domain of *apterous-Gal4* driver used to co-express *UAS-Tsc1* and *UAS-Tsc2*. (C, C') *tsc1^Q87X^* homozygous mutant MARCM clones located at the wing primordium. (D, D') MARCM, M^+^ *akt^q^, tsc1^Q87X^* homozygous mutant clones located at the wing primordium. Mutant clones are marked by the expression of GFP (green) in A, C and D. A'- D' show P-dAkt channels only, the compartment boundary (B') or the boundaries of the clones (A', C', D') are traced with a white line. Genotypes: (A, A') *hs-FLP, UAS-GFP^nuc^, tub-Gal4/+; FRT82B, akt^q^*/*FTR82B, tub-Gal80, M*. (B, B') *yw; ap-Gal4*/+, *UAS-Tsc1, UAS-Tsc2*/+. (C, C') *hs-Flp, UAS-CD8::GFP; tub-Gal4/+; FRT82B, tsc1^Q87X^/FRT82B, tub-Gal80*. (D, D') *hs-FLP, UAS-GFP^nuc^, tub-Gal4/+; FRT82B, akt^q^*, *tsc1^Q87X^/FTR82B, tub-Gal80, M*.

Cell autonomous regulation of dAkt phosphorylation by direct negative feedback has to date not been shown to occur *in vivo*. To test whether the negative feedback on dAkt phosphorylation also occurs *in vivo*, i.e. during *Drosophila* development, we used the wing imaginal disc of the third instar larva. We took advantage of the unique features of the *akt^q^* allele [72], a loss of function allele that encodes a kinase-inactive dAkt protein due to mutation of the DF^327^G motif in kinase subdomain VII into DI^327^G. This dAkt mutant protein is unable to phosphorylate downstream components, but is readily expressed and can be phosphorylated by upstream signaling components [72]. We generated homozygous mutant *akt^q^* clones in the wing imaginal discs using FLP-FRT-mediated mitotic recombination using the MARCM technique [73], [74]. This confers GFP expression to the cells expressing the mutant allele only. dAkt protein expression in *akt^q^* mutant clones and in the neighboring cells expressing wild-type dAkt were at similar levels (Figure S3). We then visualized dAkt phosphorylation of *akt^q^* mutant and wild-type cells by immunofluorescence using the anti P-dAkt antibody. The presence of an inhibitory mechanism that depends on the activity of the dAkt kinase and negatively feeds back on dAkt phosphorylation predicts enhanced P-dAkt levels in *akt^q^* mutant cells when compared to cells expressing wild-type dAkt. Consistently, we observed drastically enhanced phosphorylation of dAkt in clones homozygous for the *akt^q^* mutation (Figure 4A). The increased dAkt phosphorylation in *akt^q^* mutant cells thus indicates that inactivation of the dAkt kinase function removes repression on dAkt phosphorylation by negative feedback. It further demonstrates the cell autonomous presence of this regulatory loop in imaginal wing discs of third instar larvae.

### Regulation of dAkt phosphorylation by the Tsc1/Tsc2 tumor suppressor complex

Having established that feedback inhibition leads to repression of dAkt phosphorylation *in vivo*, we asked whether changes in Tsc1/Tsc2 function would affect the feedback activity on dAkt phosphorylation (Figure 4). dAkt-mediated phosphorylation of Tsc2 inhibits the function of the Tsc1/Tsc2 tumor suppressor complex [63], [75]. First, we co-expressed Tsc1 and Tsc2 in the dorsal compartment of the third instar imaginal wing disc under the control of ap-Gal4. If the Tsc1/Tsc2 complex defines the feedback inhibition of dAkt phosphorylation *in vivo*, overexpression of Tsc1/Tsc2 is expected to result in increased dAkt phosphorylation. Indeed, compared to ventral control cells, dAkt phosphorylation is clearly elevated in dorsal cells (Figure 4B). This result indicates that forced Tsc1/Tsc2 expression represses the feedback inhibition. Conversely, we induced homozygous mutant clones of either *tsc1^Q87X^* or *tsc2^192^*, resulting in the lack of functional Tsc1/Tsc2 tumor suppressor complex [76], [77], [78]. Complementary to the Tsc1/Tsc2 overexpression experiment, we expected a decrease in dAkt phosphorylation. Indeed, we found reduced dAkt phosphorylation levels in *tsc1^Q87X^* homozygous mutant cells, when compared to wild-type control cells (Figure 4C).

Next, we addressed whether the dAkt feedback signaling is routed from dAkt to the Tsc1/Tsc2 complex by evaluating the P-dAkt immunoreactivity in *akt^q^*, *tsc1^Q87X^* double mutant clones. If Tsc1/Tsc2 transduces the feedback signal originating from dAkt, we expect that the additional elimination of *tsc1* function in an *akt^q^* clone reverses the increased dAkt phosphorylation found in a clone of *akt^q^* single mutant cells. Consistent with this prediction, the level of dAkt phosphorylation in *akt^q^*, *tsc1^Q87X^* double mutant clones was not elevated when compared to cells with wild-type expression of Tsc1/Tsc2 and dAkt. To the contrary, P-dAkt levels were decreased, more like cells singly deficient in *tsc1* function (Figure 4C). We therefore conclude that the Tsc1/Tsc2 tumor suppressor complex controls dAkt phosphorylation *in vivo* by defining the feedback inhibition.

### Negative feedback regulation of dAkt phosphorylation is independent of FoxO

Transcription factors of the FoxO family have emerged as central mediators of the PI3K-dAkt signal transduction pathway [21], [79]. In *Drosophila* cell culture and the adult fly, the *InR* transcript is selectively transcribed and translated under conditions of low dAkt signaling levels [38], [39], [40]. Since these data suggests an alternative route of feedback mediated regulation of dAkt phosphorylation that is independent of TORC1 and S6K, we wanted to test the role of dFOXO in the negative feedback regulation of dAkt phosphorylation in the third instar imaginal wing disc. To do so, we used an activated form of dFOXO (dFOXO-TM), in which the dAkt phosphorylation sites have been replaced by alanines (Figure S4) [80]. These mutations result in constitutively nuclear localization of dFOXO, and strong transactivation of target genes both *in vitro* and *in vivo* [38], [39], [81]. Expression of dFOXO-TM by means of ap-Gal4 did not reveal any discernable differences in dAkt phosphorylation between dFOXO-TM expressing versus non-expressing cells (Figure S4A). Furthermore, mitotic clones homozygous for the *dfoxo^25^* loss of function allele, which is predicted to encode a truncated protein [81], retain a similar amount of P-dAkt as wild-type control cells (Figure S4B). Finally, *akt^q^, dfoxo^25^* double mutant clones show increased dAkt phosphorylation, similar to homozygous clones of *akt^q^* alone (Figure S4C). These data indicate that dFOXO is not involved in the negative feedback regulation of dAkt phosphorylation in the developing wing disc.

### Tsc1/Tsc2 regulates dAkt phosphorylation via TORC1

**Figure 5:**
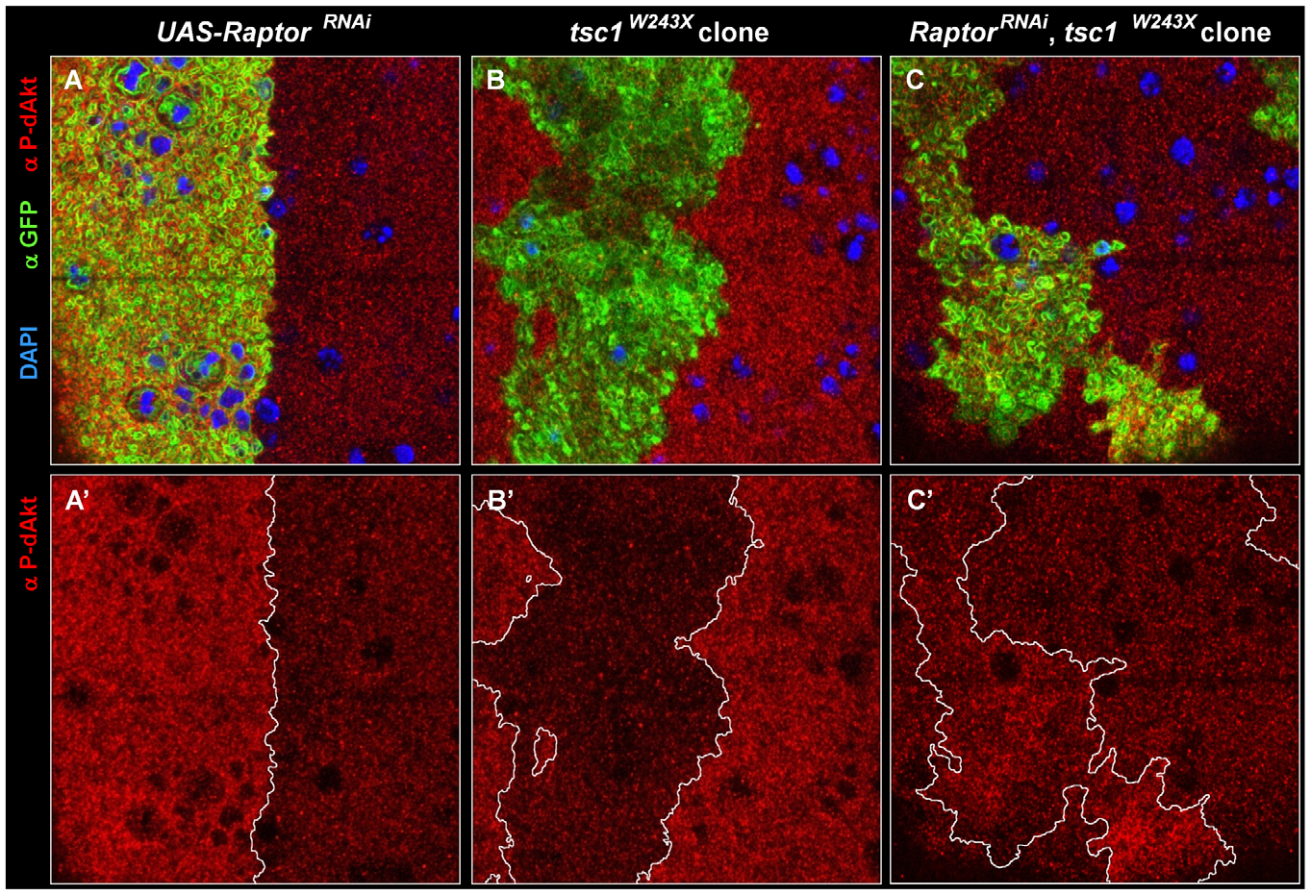
Epistatic relation of TORC1 to Tsc1. (A-C') Single tangential optical sections of third instar wing imaginal discs stained with DAPI (A-C, blue), anti P-dAkt (A-C, A'-C' red) and anti-GFP (A-C, green). (A, A') View of the dorso-ventral boundary located on the wing primordium. GFP expression (green) marks the expression domain of *apterous-Gal4* driver and the *UAS-raptor^RNAi^* hairpin expression construct. (B, B') *tsc1^W243X^* homozygous mutant clone located at the wing primordium. (C, C') Homozygous mutant clone of *tsc1^W243X^* simultaneously expressing *UAS-raptor^RNAi^* located on the wing primordium. Mutant clones are marked by the expression of GFP (green) in B and C. A', B' and C' show P-dAkt channel only, the compartment boundary (A') or the boundaries of the clones (B', C') are traced by a white line. Genotypes: (A, A') *yw; ap-Gal4*/+, *UAS-raptor^RNAi^/+*. (B, B') *hs-Flp, UAS-CD8::GFP; tub-Gal4/+; FRT82B, tsc1^W243X^/FRT82B, tub-Gal80*. (C,C') *hs-Flp, UAS-CD8::GFP; tub-Gal4/+;UAS-raptor^RNAi^,FRT82B, tsc1^W243X^/FRT82B, tub-Gal80*.

To further delineate the feedback inhibition pathway mediated by the Tsc1/Tsc2 complex, we analyzed the requirement of TORC1 in the downregulation of dAkt phosphorylation. The protein kinase TOR has been found in close physical proximity of Tsc1 and Tsc2, and biochemical and genetic evidence have established that TOR is a central mediator of Tsc1/Tsc2 signaling [76], [82], [83], [84], [85]. However, TOR is part of TORC1 as well as TORC2, and the former is required for S6K activation, which, in cell culture, brings forth negative feedback on dAkt phosphorylation, while the latter is required for hydrophobic motif phosphorylation of dAkt [16]. To interfere specifically with TORC1 function, we expressed an RNAi hairpin construct against Raptor (*raptor^RNAi^*), a component present only in TORC1 and not in TORC2 [49], [86]. Using ap-Gal4, we compared the dAkt phosphorylation levels in *UAS-Raptor^RNAi^* expressing, GFP-positive dorsal cells to those in wild-type, GFP-negative control cells in the ventral compartment (Figure 5A). If TORC1 is required to drive feedback inhibition of dAkt phosphorylation, its inactivation should augment the level of P-dAkt immunoreactivity. Accordingly, we observed increased P-dAkt staining in *Raptor^RNAi^* cells.

We further tested whether TORC1 is required for the decrease in dAkt phosphorylation observed in *tsc1* mutant wing disc cells. We expressed *raptor^RNAi^* in mitotic clones homozygously mutant for *tsc1^W243X^*. Loss of *tsc1* results in derepression of Rheb and TORC1 activity [23], [64], which, accordingly, resulted in excessive feedback inhibition of dAkt phosphorylation in *tsc1* mutant cells (Figure 5B). Feedback inhibition by Tsc1/Tsc2 mediated through TORC1 predicts that loss of *tsc1* concomitant with a reduction of *raptor* function by RNAi confers the same P-dAkt phenotype as that of *raptor^RNAi^* alone. Indeed, *raptor^RNAi^* expression in *tsc1^W243X^* mutant cells displayed an increase in P-dAkt immunostaining, as seen in cells expressing *raptor^RNAi^* alone (Figure 5C). This is consistent with the loss of negative feedback inhibition of dAkt phosphorylation in *raptor^RNAi^*, *tsc1^W243X^* cells, indicating an epistatic relationship of TORC1 to Tsc1 in the negative feedback circuit.

### Two distinct modes of inhibitory feedback signaling to dAkt

**Figure 6:**
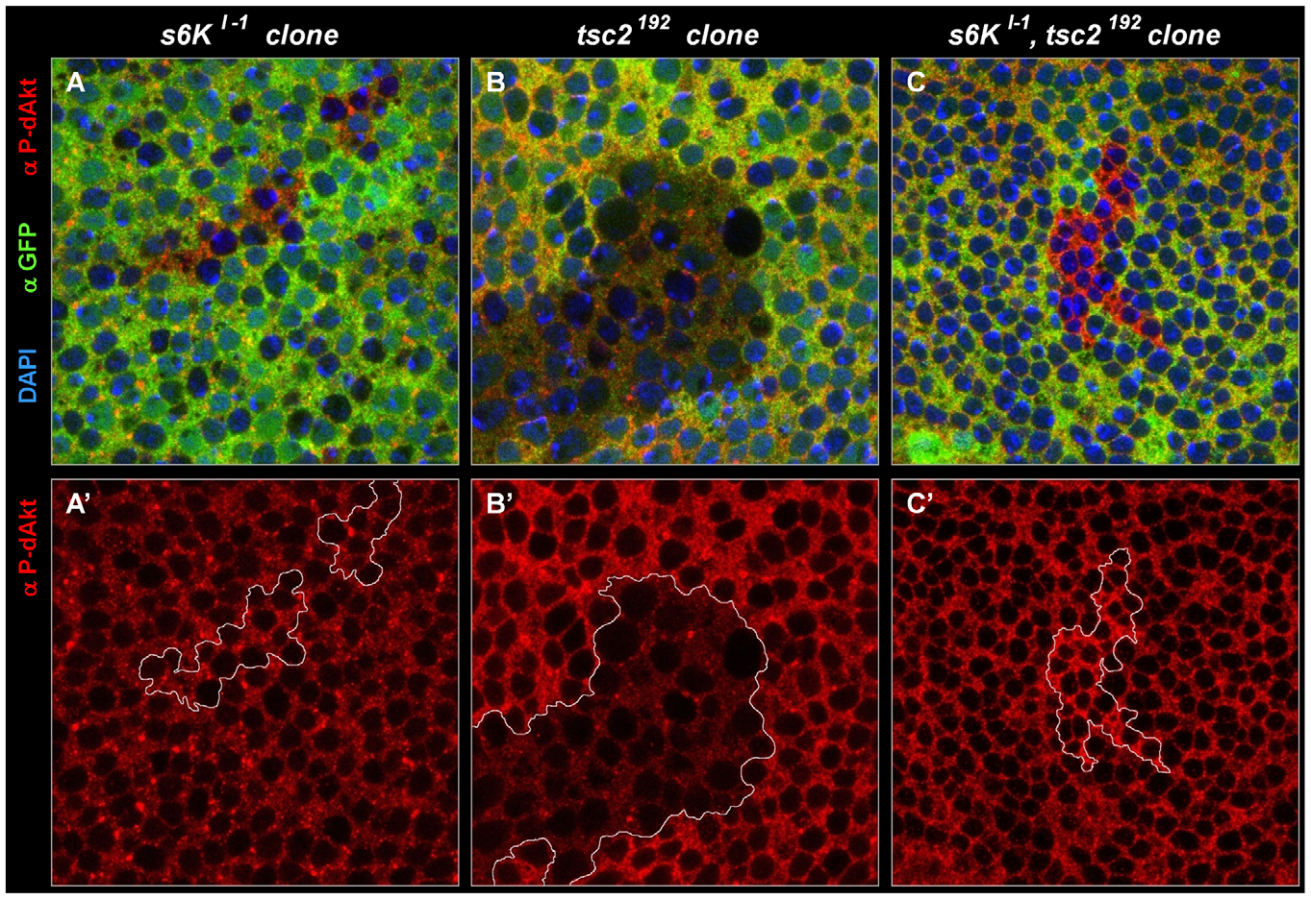
Negative feedback to Akt is S6K–independent in a wild-type background, and S6K–dependent in a *tsc2* mutant background. (A-C') Single tangential optical sections of third instar wing imaginal discs stained with DAPI (A-C, blue), anti P-dAkt (A-C, A'-C', red) and anti-GFP (A-C, green). All mutant clones shown here are marked by the absence of GFP (green) and are traced by a white line (A'-C'). (A, A') *s6K^l-1^* homozygous mutant clone located at the wing primordium. (B, B') *tsc2^192^* homozygous mutant clone. (C, C') Homozygous clone simultaneously mutant for *tsc2^192^* and *s6K^l-1^*. A', B' and C' show P-dAkt channel (red) only. Genotypes: (A, A') *hs-Flp/+; s6K^l-1^, FRT80B/ubi-GFP, FRT80B*. (B, B') *hs-Flp; tsc2^192^, FRT80B/ubi-GFP, FRT80B*. (C,C') *hs-Flp; s6K^l-1^, tsc2^192^, FRT80B/ubi-GFP, FRT80B.*

S6K is a central player downstream of TORC1, and TORC1 activity is directly required for the activation of S6K [25], [35]. To test the role of S6K in the feedback inhibition of dAkt phosphorylation, we generated homozygous clones of an *s6K* null allele (*s6K^l-1^*) [24], and investigated the level of dAkt phosphorylation in reference to wild-type tissue. If S6K mediates the feedback inhibition emanating from TORC1, dAkt phosphorylation should be increased in *s6K^l-1^* cells, since the inhibitory feedback on dAkt would be released. To our surprise, no change in P-dAkt level was apparent (Figure 6A), suggesting that S6K does not regulate the negative feedback signaling mediated by Tsc1/Tsc2 and TORC1 at this stage of wing imaginal disc development. Because this *in vivo* finding differed strikingly from the results in *Drosophila* cell culture, we verified that the *s6K^l-1^* chromosome did not carry additional mutations. The lethality associated with our *s6K^l-1^* stock [24] was rescued by expressing a S6K^WT^ cDNA from an act-Gal4 driver. To further assess if the phosphorylation status of dAkt varies in the *s6K^ l-1^* mutant background in a tissue dependent fashion, we performed a western blot analysis of extracts from whole third instar *wild-type* and *s6K^l-1^* mutant larvae (Figure S5). Consistent with our result in wing imaginal disc clones of *s6K^ l-1^*, we did not observe an increase of P-dAkt. However, we detected a downregulation of total Akt protein expression in extracts from *s6K^l-1^* mutant larvae when compared to *wt*, thus suggesting additional regulatory mechanisms controlling dAkt in different tissues.

Most studies on feedback regulation of dAkt by S6K are carried out in the context of either *tsc1* or *tsc2* mutants or other experimental settings of putatively high TORC1 activity [18], [87]. This led us to probe the dependence of the feedback inhibition on S6K in a high TORC1 signaling background induced by a *tsc2* mutant context. First, we confirmed that, similar to *tsc1^Q87X^* (Figure 4C), P-dAkt is downregulated in cells homozygously mutant for *tsc2^192^* (Figure 6B). Subsequently, we generated *s6k^l-1^*, *tsc2^192^* double mutant clones and stained the cells with anti P-dAkt antiserum (Figure 6C). Surprisingly, and in contrast to *s6k^l-1^* single mutant clones and *tsc2^192^* single mutant clones, *tsc2^192^*, *s6k^l-1^* double mutant tissue of the wing imaginal disc displayed elevated P-dAkt levels compared to wild-type cells (Figure 6). We observed a similar result using a different allelic combination, s6K^l-1^, tsc2^*^ (data not shown). This result suggests that the feedback inhibition of dAkt phosphorylation depends on S6K only when TORC1 signaling is elevated above its wild-type activity.

### S6K as sensor and regulator of dAkt–TOR signaling activity

The observation that ablation of S6K function only affects feedback inhibition of dAkt phosphorylation when TORC1 signaling is elevated suggests that activation of S6K by TORC1 in a wild-type context is insufficient to affect dAkt phosphorylation. The activation of S6K by TORC1 involves phosphorylation of several sites in the auto-inhibitory domain and the linker region of S6K [88], [89]. We used ap-Gal4 to express either wild-type S6K (S6K^WT^), or mutant S6K forms (S6K^TE^, S6K^STDE^ or S6K^STDETE^ [90], which are intrinsically activated due to substitution of several serine and threonine residues by acidic amino acids in the linker (S6K^TE^), the autoinhibitory domain (S6K^STDE^), or both (S6K^STDETE^). Overexpression of S6K^WT^ did not visibly change the level of dAkt phosphorylation when compared to ventral, non-S6K-expressing control cells (Figure S6). However, expression of the activated alleles S6K^TE^, S6K^STDETE^ and, to a limited extent, S6K^STDE^, resulted in decreased dAkt phosphorylation, when compared to ventral non-expressing cells, reminiscent of the effect of high TORC1 signaling. We further addressed whether activated S6K is also sufficient to elicit inhibition of P-dAkt when TORC1 activity is low. To this end, we co-expressed Tsc1 and Tsc2, to dominantly inhibit TORC1, and assessed P-dAkt levels in the absence or presence of S6K^TE^ co-expression (Figure S7). As observed above (Figure 4), Tsc1/Tsc2 expression caused a pronounced increase in P-dAkt (Figure S7A and S7A'). Strikingly, simultaneous expression of dominant active S6K (S6K^TE^, Figure S7B and S7B') reversed the elevated P-dAkt down to a near wild-type level. Altogether, these results suggest that activation of S6K is sufficient to elicit feedback inhibition of dAkt phosphorylation under normal or inhibited TORC1 activity levels. However, in wild-type wing imaginal disc cells, endogenous S6K is not sufficiently activated to regulate the feedback inhibition of dAkt phosphorylation. These results suggest that S6K serves as a sensor and homeostatic regulator of dAkt-TOR signaling intensity *in vivo*.

## Discussion

Akt signaling provides a critical nexus between PI3K and TORC1 signaling. Excessive activation of dAkt and its effector pathways drives unrestricted cell growth and proliferation, as observed in cancers. Conversely, an impaired response of Akt to stimulating factors like insulin in peripheral tissues contributes to metabolic syndromes and diabetes mellitus. Hence, the activity of Akt-TOR signaling needs to be maintained within well-defined physiological boundaries within a critical upper as well as lower limit. We now provide the first evidence that, during development, negative feedback monitors and autoregulates the activity of the dAkt-TOR signaling pathway in wing imaginal disc tissue *in vivo*.

**Figure 7:**
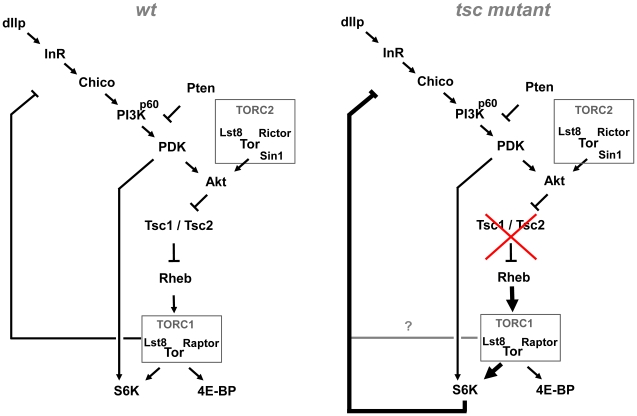
Model of the Akt–TOR signaling transduction network *in vivo*. Under wild-type conditions, negative feedback to the PI3K/Akt signaling branch is mediated by TORC1 (containing TOR, Lst8 and Raptor), independently of S6K. Under conditions of high TORC1 signaling load, induced e.g. by mutations in *tsc1* or *tsc2*, negative feedback becomes S6K dependent. See discussion for details.

We studied the control of dAkt activation in *Drosophila* Kc_167_ cells and *in vivo* using the wing disc of the *Drosophila* third instar larva. Our results highlight that dAkt centers itself in a circular pathway with two modes of negative feedback regulation (Figure 7). Consequently, the levels of dAkt phosphorylation *in vivo* are controlled not only by the input by extracellular factors into the signaling pathway, but also by the amplitude of the dAkt-TORC1 signal itself. In the wild-type context of the third instar wing disc, the negative feedback on dAkt phosphorylation is mediated by TORC1 and is S6K-independent. Interestingly, increased dAkt-TOR signaling activity switches the mechanism of negative feedback from an S6K-independent- to an S6K-dependent feedback mode (Figure 6). We interpret this finding as a rewiring of the dAkt-TOR signaling network that illustrates a dynamic response of a signaling circuit to signaling loads.

### TORC1–dependent feedback control of dAkt phosphorylation *in vivo*

Our initial experiments on dAkt phosphorylation in wing imaginal discs demonstrated that expression of activated PI3K (PI3K^CAAX^) or dominant negative InR (InR^K1409A^) elevated or repressed, respectively, the level of dAkt phosphorylation. This observation indicates that in these cells, dAkt phosphorylation can be enhanced or repressed, depending on the signaling input, and thus that dAkt has “room” for quantitative regulation of activation. Accordingly, high levels of negative feedback, caused by mutational inactivation of *tsc1* or *tsc2,* suppress dAkt phosphorylation, while low levels of negative feedback signaling, as in *Raptor^RNAi^*-expressing cells, enhance dAkt phosphorylation. These results lead to the conclusion that the wild-type level of dAkt phosphorylation in these cells is set by negative feedback regulation that is executed by the Tsc1/Tsc2-Rheb-TORC1 arm of the dAkt-TOR pathway. Interestingly, in *s6K^l-1^* mutant clones, levels of P-dAkt are unchanged, indicating the independence of the negative feedback circuit from S6K activity under these conditions. These results differ from the reported elevated Akt kinase activity in extracts from whole *s6K^l-1^* second instar larvae [65], [91]. Studies of whole larval extracts may reflect the regulation in endoreplicating tissues, which dominate the body mass at that stage of development, whereas our results of *s6K^l-1^* mutant clones in the wing disc examine Akt phosphorylation in a mitotically active tissue. The disparity in Akt phosphorylation may therefore reflect differences in negative feedback regulation of Akt at discrete stages of development and in distinct tissues. The recent observation that inhibition of TORC1 by rapamycin treatment in adult flies results in loss of S6K phosphorylation and, presumably, activity without eliciting changes in dAkt phosphorylation serves as a case in point [92]. The tissue specificity of dAkt feedback regulation will be an interesting topic of future investigation.

In the wing imaginal disc, the feedback-driven changes in dAkt phosphorylation occur in a manner that is uncoupled from changes in dAkt protein expression. Indeed, the genetic manipulations that resulted in changes in dAkt-TOR signaling activity left the dAkt expression levels unchanged. The only exception of a slight reduction in overall dAkt level, yet increased dAkt phosphorylation, was observed upon expression of PI3K^CAAX^ (see Figure S3E and S3E'). We therefore propose that, in the wing imaginal disc, a change in the phosphorylation status of dAkt, but not in protein expression, represents the relevant regulatory event *in vivo* that is targeted by TORC1-dependent negative feedback. Changes in dAkt activity by manipulating the negative feedback can have significant biological effects [42], [93], [94]. In contrast to our findings in wing discs, we do observe a reduction of Akt protein levels in whole third instar larval extracts of *s6K^l-1^* mutants. Since in third instar larvae the wing imaginal discs represent only a minor and select fraction of cells and tissues, the mass disparity of larval vs. imaginal wing tissue may explain this differing result of dAkt in our western blot vs. the clonal wing imaginal disc analysis. Of note, our western blot analyses differed significantly from those reported by Radimerki et al. [65], [91], in the use of third versus second instar larval extracts, differences in protein extraction, antibodies, and normalization against total protein versus Akt levels. We interpret the divergent results as due to different assays and developmental stages analyzed. Importantly, a change of Akt protein levels under altered Akt-TOR signaling conditions is not unprecedented [95].

Although the TORC1-dependent feedback needs to be biochemically characterized, two mechanisms may be envisioned. First, TORC1 could participate in an inhibitory step required for downregulation of dAkt activity. This possibility may be supported by experimental evidence that TORC1 can elicit a direct inhibitory phosphorylation of IRS1 in mammalian cell culture [96], [97]. Second, disruption of TORC1 by RNAi knockdown against specific components of the complex may release the remaining components of TORC1, and might shift a mass-action equilibrium between TORC1 and TORC2 towards TORC2. While such equilibrium has been suggested [98], there is, to our knowledge, only experimental evidence for an equilibrium shift towards TORC1 when TORC2 is disrupted, but not vice versa [16], [50]. Lastly, in mammalian cells the Tsc1/Tsc2 complex is required for proper TORC2 activation, independently from its role in negative feedback signaling [99]. Nevertheless, we observe that Raptor^RNAi^ hairpin expression reverses the decrease in dAkt phosphorylation in a *tsc1* mutant clone, although not to the same extent as expression of the Raptor^RNAi^ hairpin driven by ap-Gal4. These findings suggest that negative feedback is a central route of Tsc1/Tsc2 to regulate dAkt phosphorylation. However, we cannot exclude a functional role of Tsc1/Tsc2 in the activation of TORC2 [99].

### dAkt–TOR pathway feedback in *Drosophila* cell culture versus *in vivo*

We also provide evidence for S6K-dependent negative feedback inhibiting the phosphorylation of dAkt *in vivo*. The S6K-dependent mode of feedback was previously proposed based on data in mammalian or *Drosophila* cell culture. Accordingly, we observed an S6K-dependent negative feedback circuit inhibiting the phosphorylation of dAkt in *Drosophila* Kc_176_ cells [65], [66], [67]. *In vivo*, however, this mode of feedback was observed only in cells with high TORC1 signaling, and was not seen in wild-type conditions, where the negative feedback mechanism is TORC1-dependent and does not depend on S6K. We therefore propose that in the wing imaginal disc, under conditions of high TORC1 signaling, the cells switch their feedback mechanism from a TORC1-dependent mode to an S6K-dependent mechanism similar to what is observed in Kc_167_ cell culture. We suggest that the constant presence of serum, insulin and high amino acid concentrations in the cell culture medium foster high TORC1 activity, favoring the S6K dependent negative feedback route. However, it is possible that in cultured Kc_167_ cells, both feedback mechanisms are simultaneously operative. Indeed, we found that, in Kc_167_ cells, RNAi-mediated knockdown of the TORC1 component Raptor triggers a stronger increase in dAkt phosphorylation than RNAi against S6K (see Figure S2B and Figure S8).

### dAkt activity and cell size

The elevated dAkt phosphorylation observed in cells with increased Tsc1 and Tsc2 expression strongly supports a negative feedback regulation of dAkt phosphorylation by the Tsc1/Tsc2 complex *in vivo*. Nevertheless, this finding is surprising at two levels. First, dAkt has been described as a positive regulator of cell size, and *akt^q^* homozygous mutant clones show reduced cell size [100]. However, forced expression of Tsc1/Tsc2 results in reduced cell size, despite elevated dAkt phosphorylation [76], [77], [78]. The reciprocal experiment highlights the same paradox: *tsc1^Q87X^* or *tsc2^192^* mutant cells have a larger size, despite decreased dAkt phosphorylation and activity [76], [77], [78]. These results may indicate that, for cell size, dAkt's function is to regulate Tsc1/Tsc2 activity, which is supported by the fact that so far no other dAkt substrate (e.g. FoxO, Gsk3beta) has been shown to elicit a cell size defect [81]. Correspondingly, the ability of Akt1 and Akt2 deficiency to suppress H-Ras mediated oncogenesis in mouse mammary glands is overcome by inactivation of *tsc2*, again supporting the hypothesis that a central function of dAkt *in vivo* is the regulation of TORC1 activity [101]. The consistency of the data in *Drosophila* with those in mice indicates that this function of dAkt in dAkt-TOR signaling is conserved. The second surprise relates to the elevated dAkt phosphorylation in the presence of ectopic Tsc1/Tsc2 expression, which may at first seem intuitive. The circular structure predicts that increased Tsc1/Tsc2 expression should inactivate the feedback inhibition of dAkt phosphorylation by repressing TORC1, thus releasing dAkt from negative feedback regulation, hence increasing dAkt phosphorylation. However, a perfectly circular dAkt-TOR pathway predicts that increased Tsc1/Tsc2 levels should trigger high dAkt activity, which in turn should inactivate the Tsc1/Tsc2 complex by direct phosphorylation of Tsc2 [75], [102], [103]. Thus, depending on the strength of the dAkt-Tsc2 connection, dAkt phosphorylation could either remain unchanged or even be reduced. However, the dAkt-Tsc2 link might be less physiologically relevant than initially suggested [75], [104], [105], pointing to additional regulatory connections of the InR-PI3K-dAkt and Tsc1/Tsc2-Rheb-TORC1 signaling branches [20], [99], [106], [107], [108], [109]. Alternatively, overexpressed Tsc1/Tsc2 may localize to a subcellular compartment where it escapes phosphorylation by active dAkt, yet can inhibit TORC1, or the derepressed activity level of dAkt might be insufficient to effectively control overexpressed Tsc1/Tsc2.

### S6K serves as a sensor of TORC1 signaling load *in vivo*

In the developing *Drosophila* wing disc, S6K is a central mediator of TORC1 activity, especially as the fly 4E-BP1 ortholog, Thor, is not expressed [110]. Since ectopic expression of activated S6K, but not wild-type S6K, results in decreased dAkt phosphorylation, we conclude that S6K activation is sufficient to elicit a negative feedback on dAkt. In the activated S6K mutants, sites in the linker and autoinhibitory domain that are normally phosphorylated by TORC1 are replaced by phospho-mimetic acidic amino acids [90]. Our data therefore suggest that linker and the autoinhibitory domain phosphorylation of S6K may function as sensor for the TORC1 signaling load. Thus, only when TORC1 is highly active, S6K will become sufficiently phosphorylated to drive the negative feedback, a scenario that is mimicked by ectopic expression of activated S6K. Of note, the S6K^TE^ and S6K^STDETE^ phospho-mimetic mutants, which have the linker site mutation, exert a visibly stronger inhibition of dAkt phosphorylation than the S6K^SDTE^ phospho-mimetic mutant of the autoinhibitory domain only. These differences may suggest that the phosphorylation site in the linker region of S6K is the predominant site for transducing TORC1 activity. In *Drosophila* cell culture, supra-physiological levels of nutrients and amino acids may then trigger the high TORC1 activity required to drive S6K-mediated feedback on dAkt phosphorylation.

Since mTOR has recently been shown to be targeted for phosphorylation by S6K [111], it is tempting to speculate about a mechanism involving a feedback by S6K on TORC1 that then could drive the switch between TORC1- and S6K-dependent feedback inhibition of Akt phosphorylation. However, the T2446 and S2448 sites in mTor that are phosphorylated by S6K are not conserved in *Drosophila* TOR.

In conclusion, we demonstrate that dependent on TORC1 signaling load, the negative feedback signal regulating dAkt activity is dynamically routed *in vivo*. It is either independent of S6K (under “normal” TORC1 activity) or dependent on S6K (when TORC1 activity is high). Therefore, we interpret the function of S6K as a sensor of TORC1 signaling that selectively provides additional dampening of the signaling input once TORC1 is highly active. These findings predict that pharmacological tools selectively impinging on S6K activity, in the context of obesity treatment or other conditions with high S6K activity, might carry the significant risk of uncontrollable TORC1 activity.

## Material and Methods

### Cell culture and RNAi in *Drosophila* Kc_167_ cells

For 384-well plate experiments, cells were uniformly dispensed into clear bottom black 384-well plates (Corning) containing 250ng of individual, arrayed dsRNAs using a MultiDrop liquid dispenser (Thermo). 8×10^3^ cells per well in 10 ul of serum-free media per well were seeded. After 60 min of incubation, 70 ul of 10% serum-containing culture medium (Schneider's Medium, Invitrogen) per well was added. After three days of incubation at 25°C, cells were washed once and starved in 80 ul serum-free medium overnight (12 hrs). For insulin stimulation, cells were exposed to a final concentration of 387 nM bovine insulin (Sigma) for 10 min. Rapamycin was used at a final concentration of 50 nM for 4 hrs, amino acid free media (Atlanta Biologicals) was used for 8 hrs. For western blotting, six-well dishes wereused and the conditions were scaled accordingly. 10 ug of dsRNA per well was added to 1.5×10^6^ cells per well in 1 ml of serum-free media, supplemented after 60 min with 5 ml of serum-containing media. For immunofluorescence, we used eight-well chamber slides, and cells were treated as described above, using 2 ug of dsRNA per well in 100 ul of serum-free media, complemented with 500 ul of serum-containing media after 60 min. All primer sequences of the genome-wide dsRNA library are available on the website of the *Drosophila* RNAi Screening Center (www.flyrnai.org). dsRNAs were generated from PCR-derived DNA templates by T7 RNA polymerase driven run-off transcription *in vitro* (Ambion). The generic T7 promoter sequence TAATACGACTCACTATAGG was added 5′ to all gene specific primers. All gene-specific primers were designed using Primer3 [112], and conceptual PCR products were controlled against off-target effects using SnapDragon (http://flyrnai.org/cgi-bin/RNAi_find_primers.pl). Gene-specific primer sequences used: *GFP*: CAAGGGCGAGGAGCTGTT, GTCGTCCTTGAAGAAGATGGTG; *CSK*: GAGGAAGCAGACGGCAAC, GGGACTTGGGCGAATGAT; *MEKK1*: AAGTGTGTGTTGGTGCTGGA, ATCTTCGGGAGGCAGGTC; *Thread*: GCTGGACTGGCTGGATAAAC, ATTCGGGATACTGGGGAAAA; *InR*: CAGCGCGAAAACTTCAATATCTTT, TGTTTTATCCAGTCCATCGGCTAT; *Chico*: CCAAGCATAGATTTGTCATTGTGC, GATCACCAGATCCCAAGACACTTT; *PI3K92E*: GAGGCACCAGATCCAAAATC, ATACAGCCGGAAGTCGTCAA; *PI3K21B*: GCTTTATCGAGACGGACCTG, GCATCCAGCAGATTGAGGAG; *Pten*: TGTATTATGCCAAGCGGAAGA, TCAATCGTTGGAGGGTTATGA, *dAkt*: GTCCACAAATCATCCGTTCC, ACCTCCTCCACCAAAATCAA; *Tsc1*: GAGGTAAACAATACGCGATGGAAG, AACTGAACTGACTCTGCTGGTCCT; *Tsc2*: CTAGACAGTCGTCAGGTGATCGTG, ACGCGACTAAGGATTTCTTCTTCA; *S6K*: TCTGCACCAAGACACTGAGG, GCAGTATGTTCTCGGGCTTC; *Raptor*: ACCTGGGTAAGGTGATTAGCAACA, AGGTGCAGAGCTTCTTAACGTCAT; *Rheb*: GCTAGGAGTGGTATTTCGGCTTC, CCAGTGCTTTGAAATAAATGGAGA; *PDK1*: CAAGGAGAAAGCATCAGCAA, GCCTATGTAACGACCGAAAATG.

### Protein extracts and western bloting

Kc_167_ cells were rinsed once, scraped in PBS, and pelleted at low speed in a table top centrifuge. The cell pellet was lysed in standard SDS-PAGE loading buffer without dye. Extracts of third instar larvae were prepared by mechanical homogenization and lysis in 50 mM Tris, 120 mM NaCl, 30 mM NaF, 50 uM NaVO_4_, 1% Triton X100, 0.1% SDS with Complete protease inhibitor cocktail (Roche). Lysates were cleared from debris and lipids by 10 min centrifugation in a table top centrifuge. For all protein lysates, protein concentrations were determined using Protein DC Assay (Bio-Rad), and total protein concentrations of lysates were adjusted accordingly.

### Cytoblot

The Cytoblot protocol for 384 well plates used here consists of 4 steps: (1) fixation, (2) permeabilization, (3) P-dAkt staining comprising of incubations with primary and secondary antibodies, and (4) DNA staining to assess total cell numbers in each individual well. Two versions of the cytoblot were used. The “first generation” cytoblot utilized HRP-conjugated secondary antibody and chemiluminescence to detect anti P-dAkt. This protocol was applied to the non-stimulated RNAi screen. The “second generation” cytoblot employed a fluorescently labeled secondary antibody and was used for the insulin-stimulated RNAi screen. The availability of a LiCor Aerius plate reader allowed the switch from luminescence to fluorescence based detection.

(1) Fixation: Tissue culture medium from the 384 well plates was removed and cells were fixed with 6% Formaldehyde for 90 minutes, followed by three washes with 80 ul of PBS. (2) Permeabilization: 0.1% Triton X-100 in PBS for 30 minutes (40 ul per well), followed by 3 washes in 80 ul PBS for 10 min. (3) P-dAkt staining: Cells were blocked in 5% non-fat milk in PBS for 60 minutes (90 ul per well). Anti-*Drosophila* P-dAkt Ser505 primary antibody (20 ul per well, 1∶800 diluted in 5% non-fat milk, Cell Signaling Technology, Beverley, MA) was added and incubated at 4°C overnight. After 3 washes with PBS (80 ul per well, 10 min), 20 ul secondary antibody (goat anti-Rabbit AlexaFluor 680, diluted 1∶2,500, Invitrogen, for “second generation Cytoblot”, used for insulin-stimulated RNAi screens) or a 1∶1,200 dilution of goat anti-Rabbit HRP (“first generation Cytoblot”, used for insulin-stimulated RNAi screen, Jackson Laboratories), was added in 5% non-fat milk and incubated for 4 hrs, followed by 3 washes with 80 ul of PBS, 10 min and 20 ul PBS was added to each well. Signal was developed by 20 ul SuperSignal West Pico Chemiluminescent Substrate (Pierce). HRP luminescence was read in Molecular Devices plate reader. Alexa 680 fluorescence was measured using a LiCor Aerius plate reader (680/720 nm). HRP luminescence and Alexa 680 fluorescence were interpreted as amounts of P-dAkt per well. (4) DNA staining was performed with Sytox Green (Invitrogen) 1∶20,000 in PBS for 30 min (40 ul per well). After 3 additional washes with PBS, plates were filled with 20 ul PBS per well and the fluorescent value of the Sytox dye DNA stain were read in a Molecular Devices plate reader (520/560 nm). This value, referred hereafter to as nuclear fluorescence (NucFl), is interpreted as the value representing relative cell numbers per well. All liquid manipulation steps were performed using a MultiDrop liquid handling device (Thermo).

### Data analysis

All individual values quantifying amounts of P-dAkt were normalized to the cell number per well using the nuclear fluorescent value from the DNA stain. For the insulin-stimulated screen, linear regression was performed on the log2(P-dAkt/NucFl) values of each individual screen plate, and residuals from the log2(P-dAkt/NucFl) values to the regression line were calculated. All residuals of each genome-wide screen were pooled and a cell number dependent error model was developed to determine locally weighted standard deviations (SD) and averages in dependence of cell number. Z-Scores using these two parameters were calculated, expressing the deviation from the local average value in SDs. All Z-Scores were corrected against position effects by setting the Mean Z-Score of each individual well position across one genome-wide screen replicate to zero. All dsRNAs with predicted off-target effects (homologies to non-target genes of 19 bp or more) were excluded from data processing and the result file. Results from the two screening replicates were averaged and a cut-off value of +/− 2.5 applied. Due to high variation within each 384 well screening plate caused by individual 96-well source plates (each 384-well screening plate is composed of aliquots from four 96-well source plates), Z-Scores for the baseline (no insulin stimulation) genome wide RNAi screen were calculated as follows: The 384 (P-dAkt/NucFl) values of each single screening plate were decomposed into the four 96 well groups, each defined by a single source plate, and the mean and SD was set to zero and one, respectively. This step compensated these inequalities, and data were recombined to 384 well plate data sets. Mean and SD for each individual 384 well plate were calculated, averaged between screens, and a cut-off value of three SDs was applied. For non-genome-wide RNAi experiments, an external standard consisting of 768 values of non-RNAi treated cells covering the whole spectrum of cell densities was used to determine cell number dependent averages and SDs to calculate experimental Z-Scores of RNAi treated wells. All P-dAkt values of non-stimulated cells were normalized using a baseline standard curve (the average non-treated, non-stimulated experiment scores zero). For the insulin-stimulated data set, the P-dAkt values of insulin-treated cells were normalized using a standard curve derived from insulin-stimulated cells (the average non-treated, insulin-stimulated experiment scores zero).

### Antibodies

All P-dAkt indirect immunofluorescence images, Cytoblots and western blots were performed using anti-*Drosophila* P-dAkt Ser505 (Lot 1 and Lot 2, Cell Signaling Technology) using a 1∶200, 1∶800 and 1∶200 dilution, respectively. For immunofluorescence, AlexaFluor594 and AlexaFluor488 conjugated secondary antibodies against Rabbit, Mouse and Goat were used 1∶500 (Invitrogen). Western blotting was performed using HRP conjugated anti-rabbit and anti-mouse antisera (Amersham). Pan-dAkt and P-S6K Ser398 (Cell Signaling Technology) were used 1∶200. Anti-GFP was purchased from Cappel and used at 1∶4000. Mouse anti alpha-Tubulin (Sigma) was used 1∶2000 for immunofluorescence. Rabbit anti S6K was a generous gift from Mary Steward and used 1∶10,000 for western blotting.

### Immunofluorescence, confocal microscopy, and image processing

Imaginal discs and *Drosophila* Kc_167_ cells were fixed using 6% Formaldyhyde in PBS (cells 10 min at room temperature, imaginal discs at 4°C overnight), permeabilized in 0.1% Triton X-100 (10 min for cells, 2 hrs for imaginal discs) and blocked with 5% BSA in PBS (1 hr). Primary antibody incubation was performed overnight at 4°C with antibody dilutions as indicated above, using 5% BSA. After 3 washes with PBS, secondary antibody was incubated overnight in 5% BSA, followed by 3 washes in PBS. Specimens were mounted using Vectrashield mounting medium with DAPI (Vector Co.). All data were acquired using a Leica SP2 confocal microscope, a 63x lens, digital zoom factor of four, 1024×1024 pixel detector setting and processed using Adobe Photoshop software. Images of experimental and control cells were processed identically.

### Genetics

Mutant wing imaginal disc clones were generated by FLP/FRT-mediated mitotic recombination using the following chromosomes: *FRT82B, akt^q^* [72]; *FRT82B, akt^q^, tsc1^Q87X^* [76]; *FRT82B, akt^EX4^* (derived by imprecise excision from *akt^P04226^*, Bloomington Stock center) *FRT82, tsc1^Q87X^* [77]; *FRT82B, tsc1^W243X^* [77]; *FRT82B, foxo^25^* [81]; *FRT82B, foxo^25^, akt^q^* [81]; *tsc2^192^, FRT80B* [77]; tsc2*, FRT80B (generous gift from I.K. Hariharan); *s6K^l-1^, FRT80B* [24]; *s6K^l-1^, tsc2^192^, FRT80B* [71]; s6K^l-1^, tsc2*, FRT80B. Males of the respective genotypes were crossed to *y,w,hs-FLP,UAS-mCD8::GFP; tub-Gal4; FRT82B,tub-Gal80/TM6B* or *y,w,hs-FLP, ubi-GFP, FRT80B* females and larvae were heat shocked 60 hrs +/− 12 hrs after egg laying (unless otherwise specified) at 37°C for 45 min. Overexpression of PI3K^CAAX^ [113], InR^DN^ (Bloomington *Drosophila* Stock Center), Tsc1 and Tsc2 [77], Foxo™ [80], S6K^WT^ [78], S6K^TE^, S6K^STDE^ and S6K^STDETE^ [90] in the dorsal compartment of the wing imaginal disc was performed using the Gal4-UAS system [58] with *y,w; ap-Gal4,UAS-mCD8::GFP* (gift from C. Micchelli). The Raptor^RNAi^ hairpin and transgenic line was generated using the VALIUM1 vector [114] as part of the transgenic RNAi project (TRIP, http://flyrnai.org/TRiP-HOME.html).

### Gene names

Based on BLAST searches, information in the public ortholog databases InParanoid [115], and Homologene [116] published sequence homologies [117], CG3004 (Fbgn0030142), and CG10105 (Fbgn0033935) were referred to as Lst8 and Sin1 [29], [50], respectively.

## Supporting Information

Figure S1 Western blot of total extracts prepared from *Drosophila* Kc_167_ cells at base line (lanes 1-6) or insulin stimulation (lanes 7-12) treated with dsRNAs as indicated and blotted with anti Pan-dAkt, anti P-dAkt, and anti-Tubulin as loading control. Top and bottom panels of anti alpha-Tubulin western blots are loading controls for the anti P-dAkt and anti Pan-dAkt western blots, respectively. Note that lane 10 from the right, (insulin-stimulated, dPDK1 RNAi treated cells) is underloaded.
(1.35 MB TIF)

Figure S2 Genome wide screen for regulators of dAkt (Ser505) phosphorylation. (A) Cartoon of the cytoblot technique used to screen 58×384 well plates containing dsRNAs covering the entire *Drosophila* genome. Each screen was performed in duplicates. Experimental values for dAkt phosphorylation are normalized to the individual cell numbers per well determined by a DNA dye staining. See experimental procedures for details. (B, C) Ranked Z-Scores (corresponding to relative P-dAkt levels) of genome wide RNAi screens at baseline (B) and Insulin stimulation (C) with the known components of InR and Tor signaling marked in red.
(0.45 MB TIF)

Figure S3 Analysis of *in vivo* dAkt protein expression in various genetic gain- and loss-of-function backgrounds of the dAkt–TOR signaling pathway. Single tangential optical sections of third instar wing imaginal discs stained with DAPI (A-C, blue), anti Pan-dAkt (A-H, A’-H’ red) and anti-GFP (A-H, green). Mitotic clones shown in (A,A’, B,B’ and G,G’) are marked by the expression of GFP (green). Clones shown in (C,C’ and D,D) are marked by the absence of GFP (green). All other images depict *apterous-Gal4* derived co-expression of various constructs with CD8::GFP. (A,A’) Specificity control of anti Pan-dAkt. Clone of homozygously *akt^EX4^* mutant cells (*akt^EX4^* is a derivative of *akt^P04226^*, generated by imprecise excision). Note the cell autonomous loss of the Pan-dAkt antigen. (B,B’) *akt^q^* clone. (C,C’) *tsc2^192^* clone. (D,D’) *s6K^l-1^, tsc2^192^* clone. (E,E’) Expression of an activated catalytic subunit of PI3 Kinase (PI3K92E^CAAX^). Note the lower expression of dAkt in the PI3K^CAAX^ expressing compartment, accompanied by high P-dAkt levels (Figure 1). (F,F’) Ectopic expression of *Raptor^RNAi^*. (G,G’) Clone of *tsc1^W243X^* simultaneously expressing *Raptor^RNAi^*. (H,H’) Ectopic expression of S6K^STDE^. Genotypes: (A,A’): *hs-FLP, UAS-GFP^nuc^, tub-Gal4; FRT82B, akt^EX4^*/*FTR82B, tub-Gal80, M*. (B,B’): *hs-FLP, UAS-GFP^nuc^, tub-Gal4; FRT82B, akt^q^*/*FTR82B, tub-Gal80, M*. (C,C’): *hs-Flp; tsc2^192^, FRT80B/ubi-GFP, FRT80B*. (D,D’): *hs-Flp; s6K^l-1^, tsc2^192^, FRT80B/ubi-GFP, FRT80B*. (E,E’): *yw/UAS-PI3K92E^CAAX^; ap-Gal4*/+. (F,F’): *yw; ap-Gal4*/+, *UAS-raptor^RNAi^*. (G,G’): *hs-Flp, UAS-CD8::GFP; tub-Gal4/+;UAS-raptor^RNAi^,FRT82B, tsc1^W243X^/FRT82B, tub-Gal80*. (H,H’): *yw; ap-Gal4*/+,*UAS- S6K^STDE^*.
(5.87 MB TIF)

Figure S4 Negative feedback regulation of the dAkt-TOR pathway is independent of dFOXO. (A-C’) Single tangential optical sections of third instar wing imaginal discs stained with DAPI (A-C, blue), anti P-dAkt (A-C, A’-C’, red) and anti-GFP (A-C, green). (A, A’): Magnified view on the dorso-ventral boundary at the wing primordium. GFP expression (green) marks the dorsal expression domain of *apterous-Gal4* driver and the activated *UAS-dFOXO^TM^* expression construct [80]. (B, B’): homozygous *foxo^25^* loss of function MARCM clone. Homozygous cells for *foxo^25^* are marked by CD8::GFP coexpression (green). (C,C’): *foxo^25^, akt^q^* homozygous loss of function MARCM clone. Homozygous cells for *foxo^25^, akt^q^* are marked by CD8::GFP (green). D/V compartment boundary as well as borders of the clones are traced by a white line in (A’-C’). Genotypes: (A, A’) *yw; ap-Gal4*/+, *UAS-FOXO-TM*/+. (B, B’) *hs-Flp, UAS-CD8::GFP/+; tub-Gal4/+; FRT82B, foxo^25^/FRT82B, tub-Gal80*. (C,C’) *hs-Flp, UAS-CD8::GFP/+; tub-Gal4/+; FRT82B, foxo^25^, akt^q^/FRT82B, tub-Gal80*.
(6.00 MB TIF)

Figure S5 P-dAkt levels are not elevated in *s6K^l-1^* whole larval extracts. Western blot of total lysates prepared from whole third instar larvae of *wt* (left lane) and *s6K^l-1^* (right lane) genetic backgrounds. Western blots probed with anti Pan-dAkt (total Akt), anti P-dAkt, anti Pan-S6K (total S6K), anti P-S6K and anti alpha-Tubulin as loading control.
(0.42 MB TIF)

Figure S6 Activated S6K is sufficient to drive negative regulation of P-dAkt. (A-D’) Single tangential optical sections of 3^rd^ instar wing imaginal discs expressing *wild-type* and activated alleles of S6K expressed by *apterous-Gal4*. Stainings with DAPI (A-D, blue), anti P-dAkt (A-D, A’-D’, red) and anti-GFP (A-D, green) are shown. GFP expression (green) marks the expression domain of the *apterous-Gal4* driver and the various *UAS-S6K* expression constructs. A’-D’ show P-dAkt channel only, the boundary of *apterous-Gal4* expressing vs. non-expressing cells are marked with by a white line. Genotypes: (A,A’) *yw; ap-Gal4/+, UAS-S6K^WT^*, (B, B’) *yw; ap-Gal4/+, UAS-S6K^TE^* (substitution Thr398Glu in the linker region). (C, C’) *yw; ap-Gal4/+, UAS-S6K^STDETE^* (combined substitutions Thr398Glu in the linker region and Ser418Asp and Thr422Glu in the autoinhibitory domain). (D,D’) *yw; ap-Gal4/+, UAS-S6K^STDE^* (substitutions Ser418Asp and Thr422Glu in the autoinhibitory domain).
(4.83 MB TIF)

Figure S7 Dominant active S6K is sufficient to inhibit P-dAkt under low TORC1 activity. (A-B’) Single tangential optical sections of 3^rd^ instar wing imaginal discs co-expressing Tsc1, Tsc2 and CD8::GFP (A, A’); and Tsc1, Tsc2, CD8::GFP and a constitutively activated allele of S6K (S6K^TE^) (B, B’). Expression of the transgenes is driven by *apterous-Gal4*. Staining with DAPI (A-B, blue), anti P-dAkt (A-B’, red) and anti-GFP (A, B, green) are shown. GFP expression (green) marks the expression domain of the *apterous-Gal4* driver and the of the various expression constructs used. A’ and B’ show the P-dAkt channel only, the boundary of *apterous-Gal4* expressing vs. non-expressing cells are marked with by a white line. Genotypes: (A, A’) *yw*; *UAS-CD8::GFP, ap-Gal4/+; UAS-Tsc1, UAS-Tsc2/+*. (B, B’) *yw*; *UAS-CD8::GFP, ap-Gal4/UAS-S6K^TE^; UAS-Tsc1, UAS-Tsc2/+*.
(4.16 MB TIF)

Figure S8 Raptor and S6K dependent negative feedback on P-dAkt. (A) Single confocal section of *S6K*, (B) *Raptor*, (C) *Luciferase* and (D) *Pten* RNAi treated *Drosophila* Kc_167_ cells stained with DAPI (blue) anti P-dAkt (green) after 10 minutes of insulin stimulation. Images were recorded and processed using identical conditions. Note the highest level of anti P-dAkt signal in the *Raptor* dsRNA treated cells.
(2.64 MB TIF)

Table S1 Amplicons identified in the RNAi screens that enhance or suppress P-dAkt levels. Averaged Z-Scores from the two screen replicates of the baseline (no stimulation) and insulin-stimulated screens are shown. The DRSC amplicon identifies individual dsRNAs from the genome wide dsRNA set. Primer and sequence information available at www.flyRNAi.org. With the exception of the InR pathway components, the hits indicated in this Table were identified using a single dsRNA and therefore need further validation to eliminate false positives. Fbgn: Fly base gene number.
(0.03 MB PDF)
