# Supplementary figures and images for: Dynamic Switch of Negative Feedback Regulation in Drosophila Akt–TOR Signaling
Source: PLoS Genet. 2010 Jun 17;6(6):e1000990. doi: 10.1371/journal.pgen.1000990 (PMC2887466; doi:10.1371/journal.pgen.1000990)

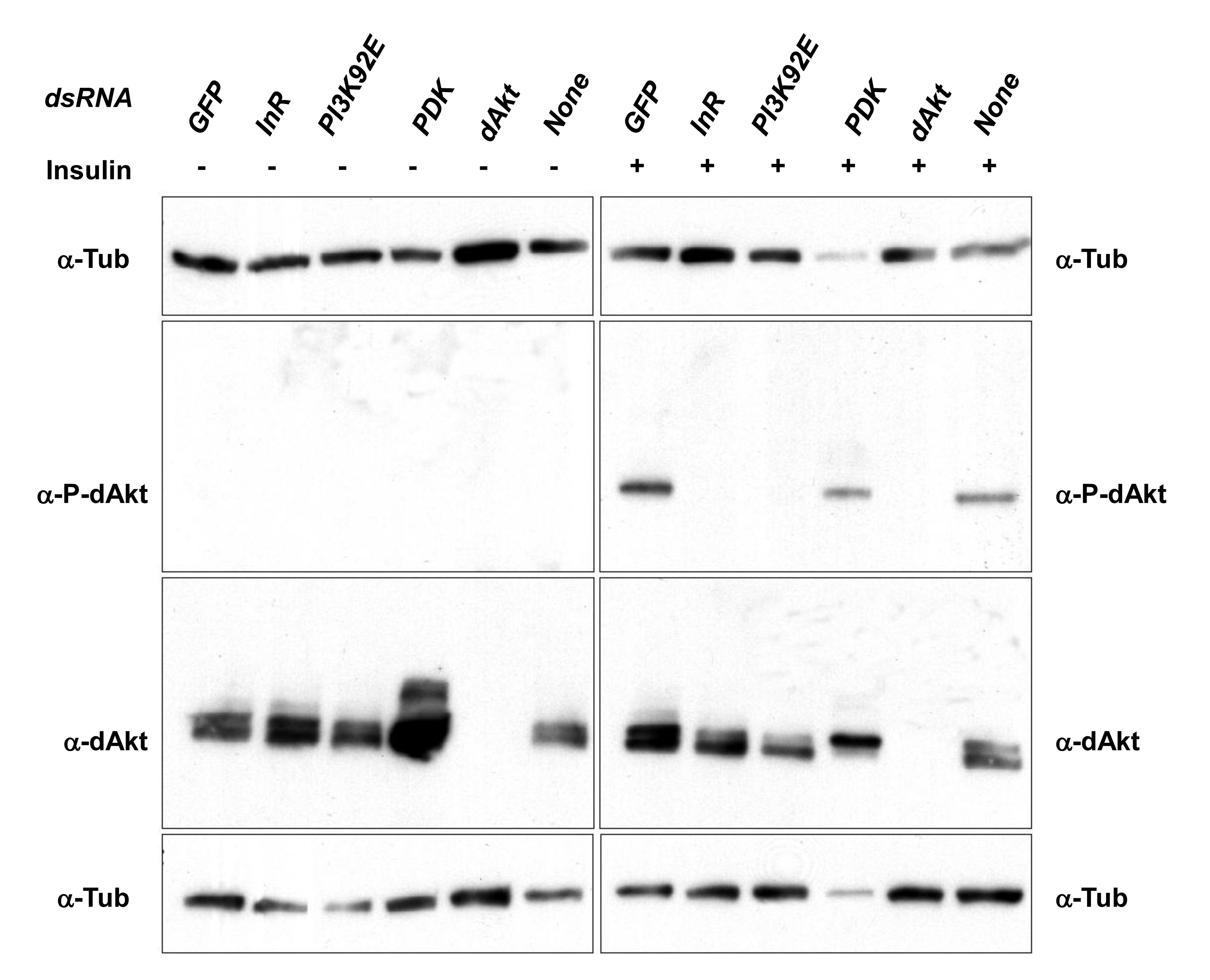

Supplement: Figure S1 — Western blot of total extracts prepared from Drosophila Kc167 cells at base line (lanes 1-6) or insulin stimulation (lanes 7-12) treated with dsRNAs as indicated and blotted with anti Pan-dAkt, anti P-dAkt, and anti-Tubulin as loading control. Top and bottom panels of anti alpha-Tubulin western blots are loading controls for the anti P-dAkt and anti Pan-dAkt western blots, respectively. Note that lane 10 from the right, (insulin-stimulated, dPDK1 RNAi treated cells) is underloaded. (1.35 MB TIF) [file pgen.1000990.s001.tif]

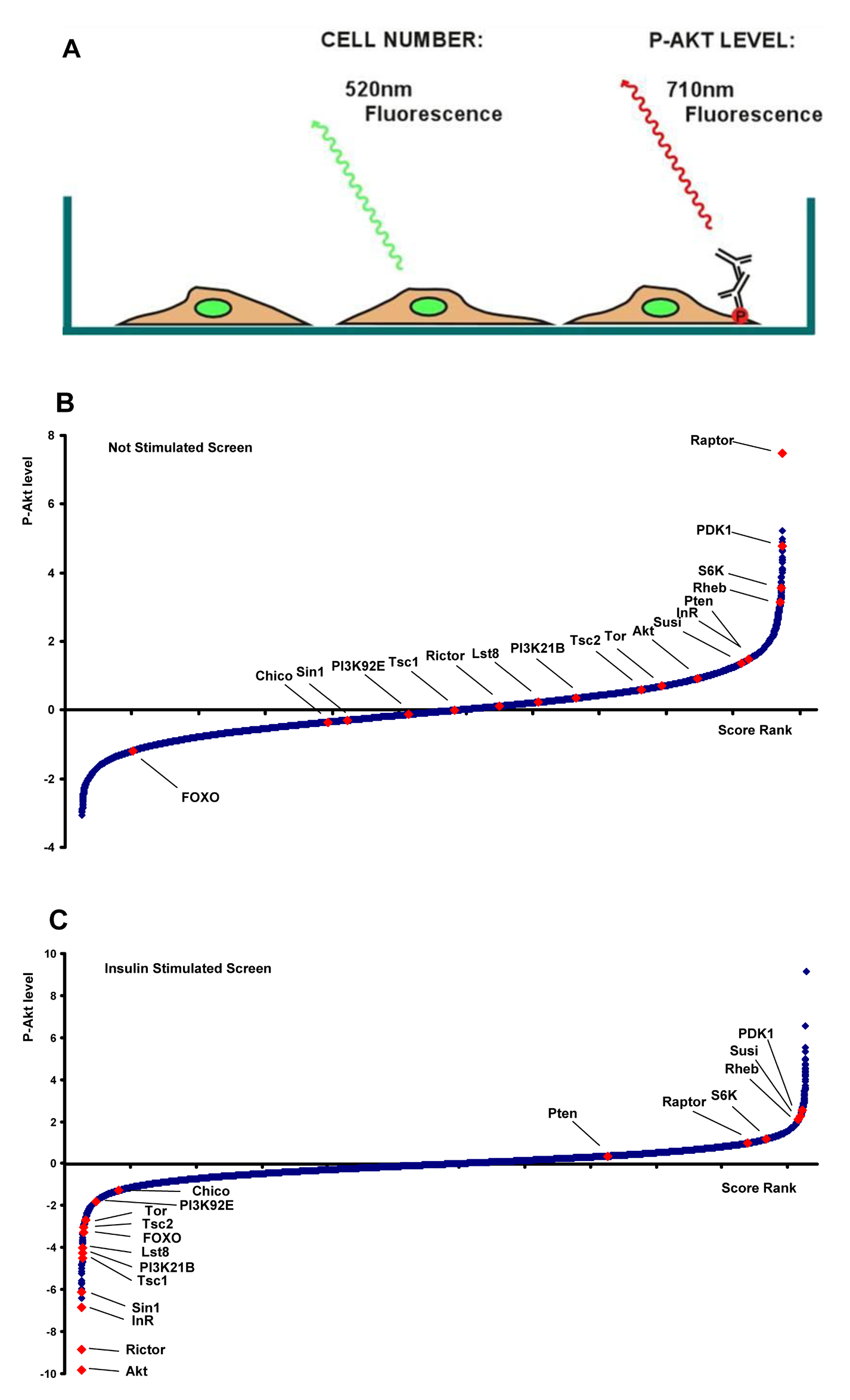

Supplement: Figure S2 — Genome wide screen for regulators of dAkt (Ser505) phosphorylation. (A) Cartoon of the cytoblot technique used to screen 58×384 well plates containing dsRNAs covering the entire Drosophila genome. Each screen was performed in duplicates. Experimental values for dAkt phosphorylation are normalized to the individual cell numbers per well determined by a DNA dye staining. See experimental procedures for details. (B, C) Ranked Z-Scores (corresponding to relative P-dAkt levels) of genome wide RNAi screens at baseline (B) and Insulin stimulation (C) with the known components of InR and Tor signaling marked in red. (0.45 MB TIF) [file pgen.1000990.s002.tif]

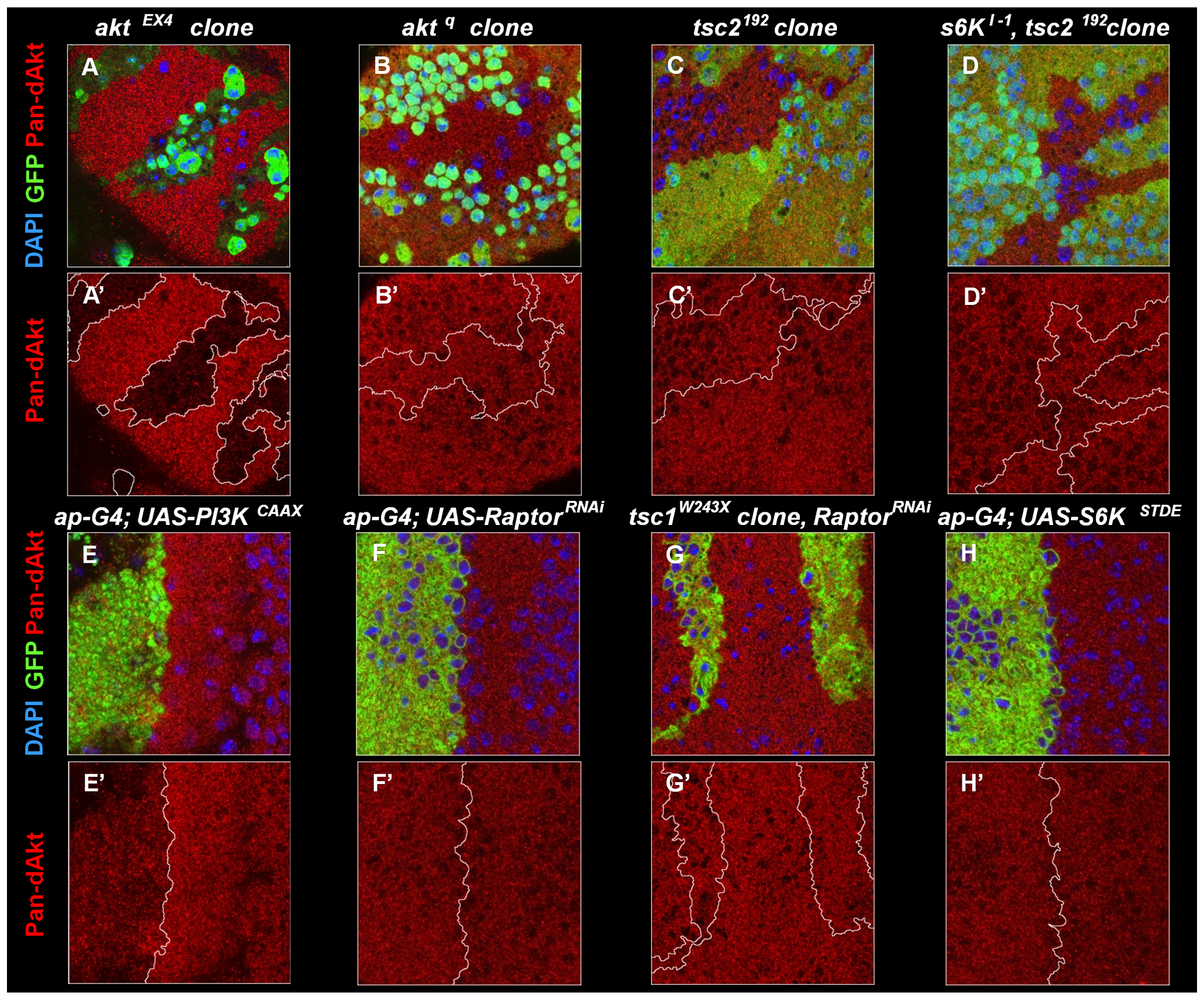

Supplement: Figure S3 — Analysis of in vivo dAkt protein expression in various genetic gain- and loss-of-function backgrounds of the dAkt–TOR signaling pathway. Single tangential optical sections of third instar wing imaginal discs stained with DAPI (A-C, blue), anti Pan-dAkt (A-H, A’-H’ red) and anti-GFP (A-H, green). Mitotic clones shown in (A,A’, B,B’ and G,G’) are marked by the expression of GFP (green). Clones shown in (C,C’ and D,D) are marked by the absence of GFP (green). All other images depict apterous-Gal4 derived co-expression of various constructs with CD8::GFP. (A,A’) Specificity control of anti Pan-dAkt. Clone of homozygously aktEX4 mutant cells (aktEX4 is a derivative of aktP04226, generated by imprecise excision). Note the cell autonomous loss of the Pan-dAkt antigen. (B,B’) aktq clone. (C,C’) tsc2192 clone. (D,D’) s6Kl-1, tsc2192 clone. (E,E’) Expression of an activated catalytic subunit of PI3 Kinase (PI3K92ECAAX). Note the lower expression of dAkt in the PI3KCAAX expressing compartment, accompanied by high P-dAkt levels (Figure 1). (F,F’) Ectopic expression of RaptorRNAi. (G,G’) Clone of tsc1W243X simultaneously expressing RaptorRNAi. (H,H’) Ectopic expression of S6KSTDE. Genotypes: (A,A’): hs-FLP, UAS-GFPnuc, tub-Gal4; FRT82B, aktEX4/FTR82B, tub-Gal80, M. (B,B’): hs-FLP, UAS-GFPnuc, tub-Gal4; FRT82B, aktq/FTR82B, tub-Gal80, M. (C,C’): hs-Flp; tsc2192, FRT80B/ubi-GFP, FRT80B. (D,D’): hs-Flp; s6Kl-1, tsc2192, FRT80B/ubi-GFP, FRT80B. (E,E’): yw/UAS-PI3K92ECAAX; ap-Gal4/+. (F,F’): yw; ap-Gal4/+, UAS-raptorRNAi. (G,G’): hs-Flp, UAS-CD8::GFP; tub-Gal4/+;UAS-raptorRNAi,FRT82B, tsc1W243X/FRT82B, tub-Gal80. (H,H’): yw; ap-Gal4/+,UAS- S6KSTDE. (5.87 MB TIF) [file pgen.1000990.s003.tif]

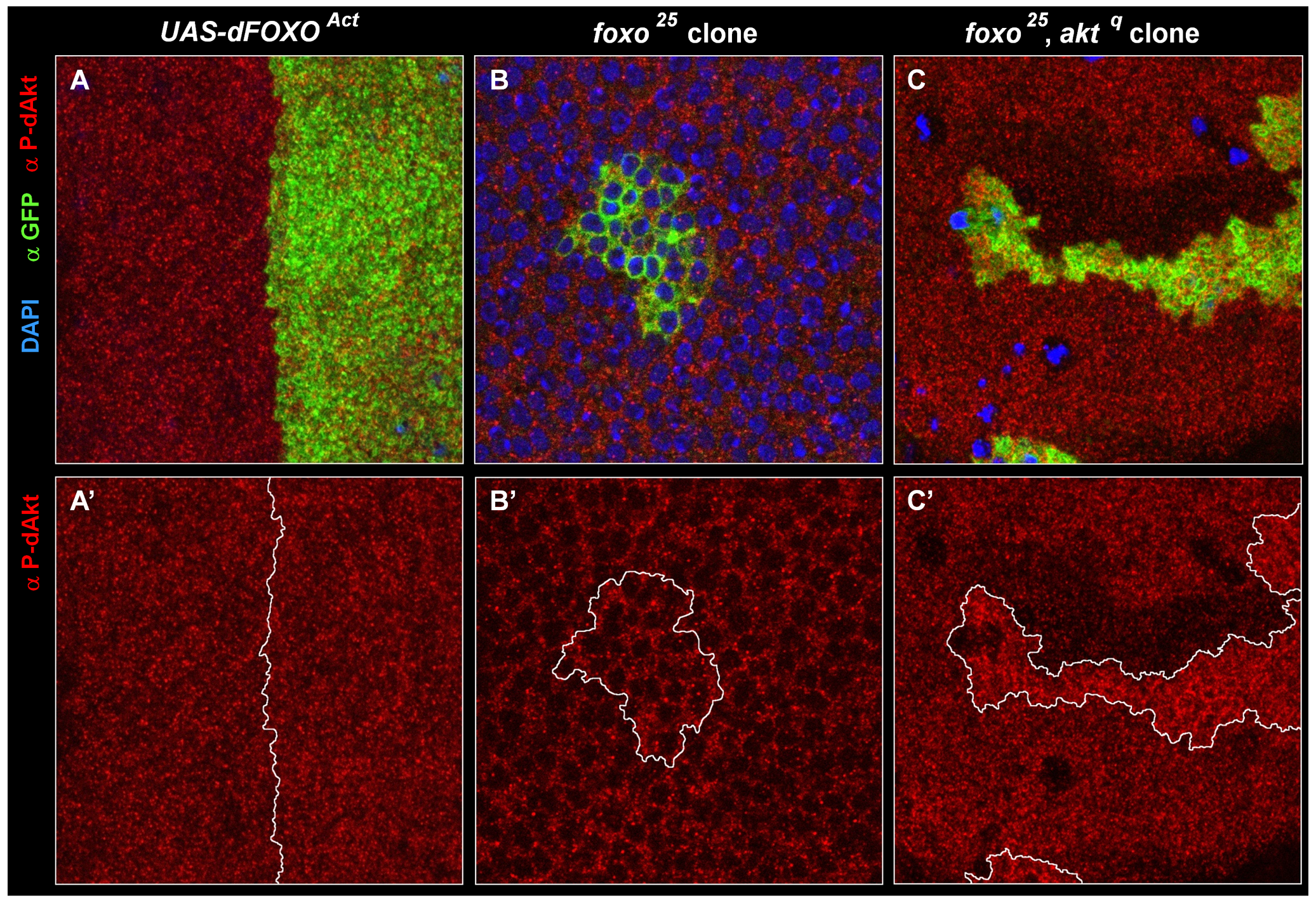

Supplement: Figure S4 — Negative feedback regulation of the dAkt-TOR pathway is independent of dFOXO. (A-C’) Single tangential optical sections of third instar wing imaginal discs stained with DAPI (A-C, blue), anti P-dAkt (A-C, A’-C’, red) and anti-GFP (A-C, green). (A, A’): Magnified view on the dorso-ventral boundary at the wing primordium. GFP expression (green) marks the dorsal expression domain of apterous-Gal4 driver and the activated UAS-dFOXOTM expression construct [80]. (B, B’): homozygous foxo25 loss of function MARCM clone. Homozygous cells for foxo25 are marked by CD8::GFP coexpression (green). (C,C’): foxo25, aktq homozygous loss of function MARCM clone. Homozygous cells for foxo25, aktq are marked by CD8::GFP (green). D/V compartment boundary as well as borders of the clones are traced by a white line in (A’-C’). Genotypes: (A, A’) yw; ap-Gal4/+, UAS-FOXO-TM/+. (B, B’) hs-Flp, UAS-CD8::GFP/+; tub-Gal4/+; FRT82B, foxo25/FRT82B, tub-Gal80. (C,C’) hs-Flp, UAS-CD8::GFP/+; tub-Gal4/+; FRT82B, foxo25, aktq/FRT82B, tub-Gal80. (6.00 MB TIF) [file pgen.1000990.s004.tif]

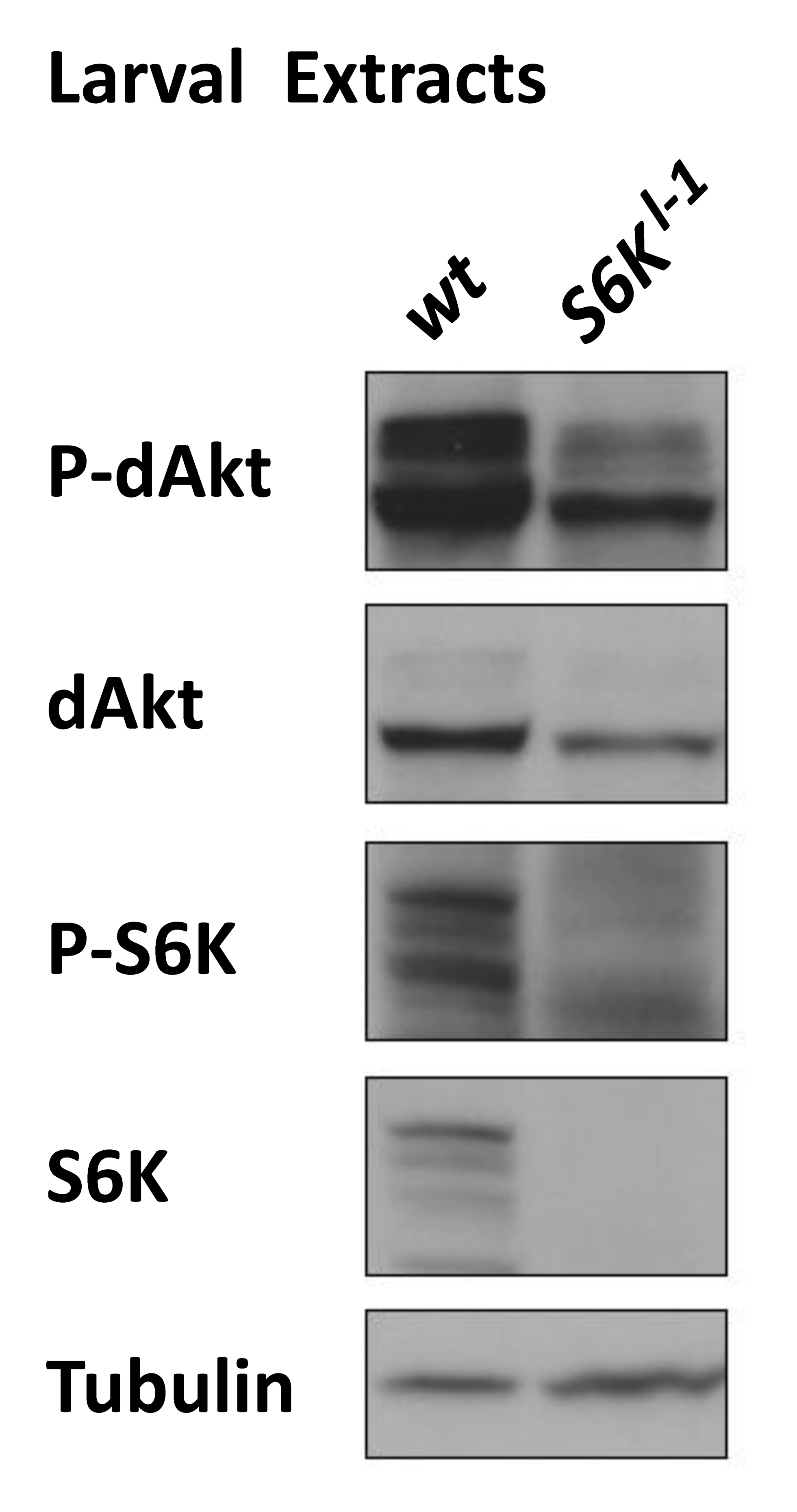

Supplement: Figure S5 — P-dAkt levels are not elevated in s6Kl-1 whole larval extracts. Western blot of total lysates prepared from whole third instar larvae of wt (left lane) and s6Kl-1 (right lane) genetic backgrounds. Western blots probed with anti Pan-dAkt (total Akt), anti P-dAkt, anti Pan-S6K (total S6K), anti P-S6K and anti alpha-Tubulin as loading control. (0.42 MB TIF) [file pgen.1000990.s005.tif]

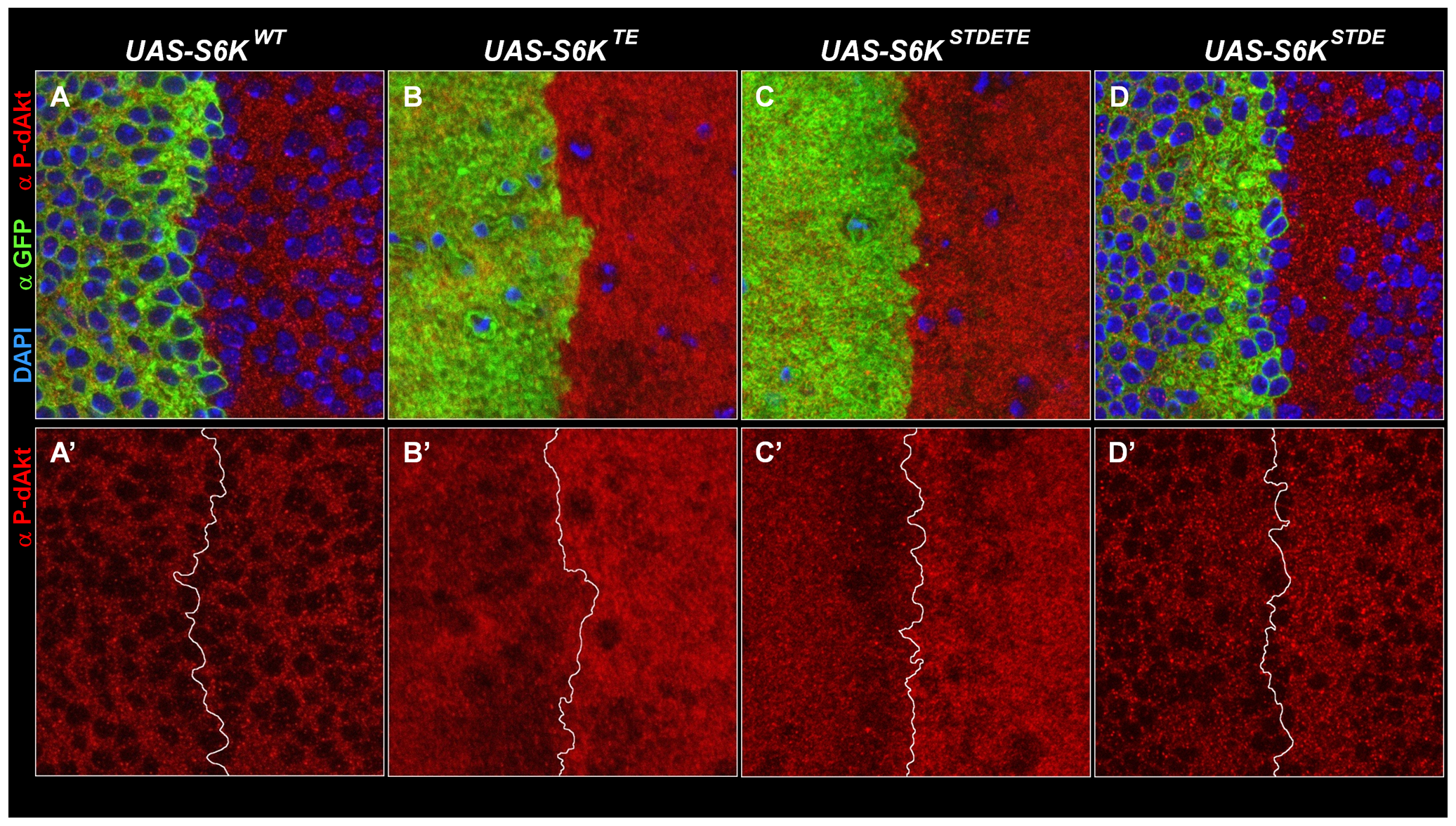

Supplement: Figure S6 — Activated S6K is sufficient to drive negative regulation of P-dAkt. (A-D’) Single tangential optical sections of 3rd instar wing imaginal discs expressing wild-type and activated alleles of S6K expressed by apterous-Gal4. Stainings with DAPI (A-D, blue), anti P-dAkt (A-D, A’-D’, red) and anti-GFP (A-D, green) are shown. GFP expression (green) marks the expression domain of the apterous-Gal4 driver and the various UAS-S6K expression constructs. A’-D’ show P-dAkt channel only, the boundary of apterous-Gal4 expressing vs. non-expressing cells are marked with by a white line. Genotypes: (A,A’) yw; ap-Gal4/+, UAS-S6KWT, (B, B’) yw; ap-Gal4/+, UAS-S6KTE (substitution Thr398Glu in the linker region). (C, C’) yw; ap-Gal4/+, UAS-S6KSTDETE (combined substitutions Thr398Glu in the linker region and Ser418Asp and Thr422Glu in the autoinhibitory domain). (D,D’) yw; ap-Gal4/+, UAS-S6KSTDE (substitutions Ser418Asp and Thr422Glu in the autoinhibitory domain). (4.83 MB TIF) [file pgen.1000990.s006.tif]

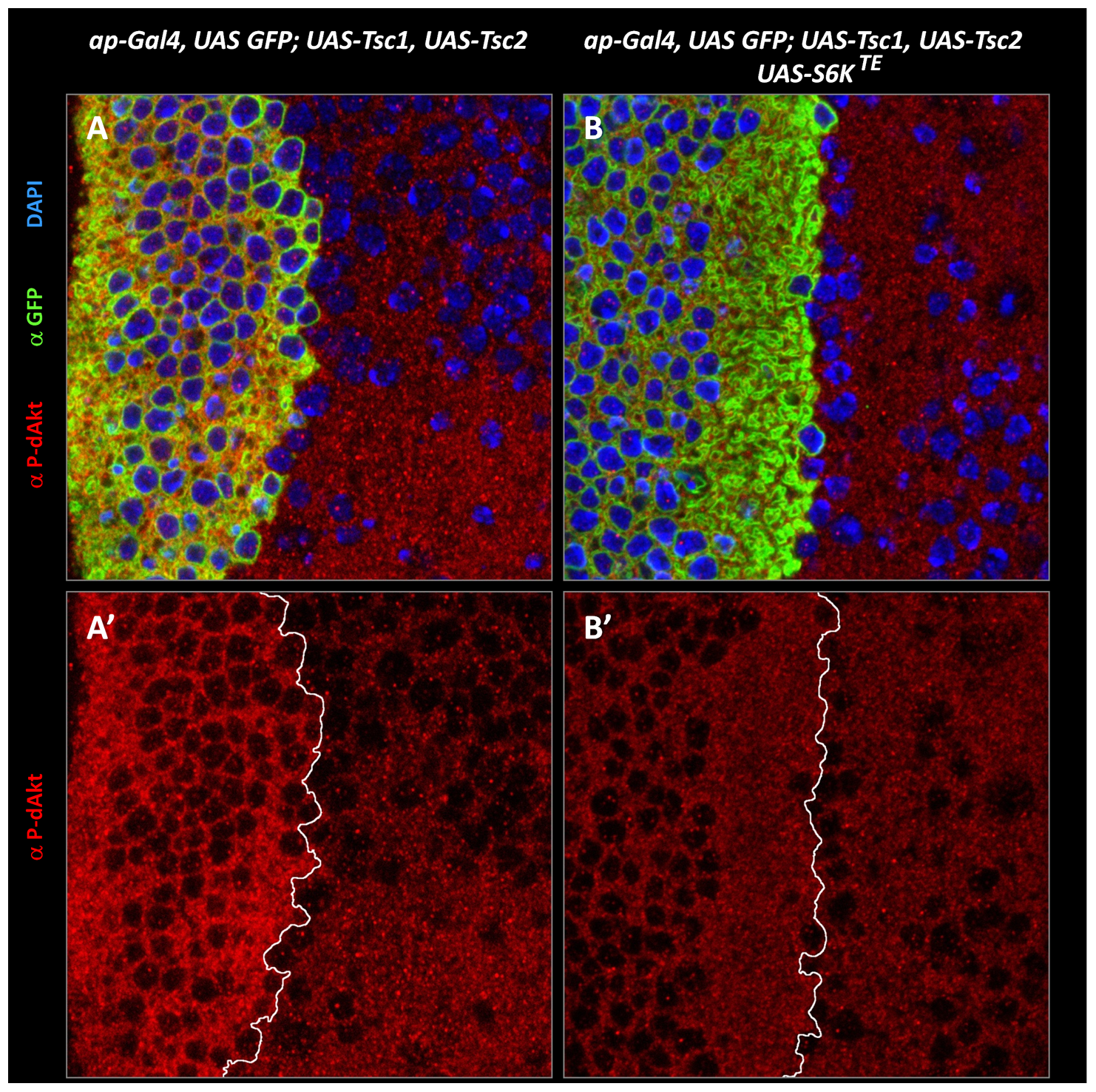

Supplement: Figure S7 — Dominant active S6K is sufficient to inhibit P-dAkt under low TORC1 activity. (A-B’) Single tangential optical sections of 3rd instar wing imaginal discs co-expressing Tsc1, Tsc2 and CD8::GFP (A, A’); and Tsc1, Tsc2, CD8::GFP and a constitutively activated allele of S6K (S6KTE) (B, B’). Expression of the transgenes is driven by apterous-Gal4. Staining with DAPI (A-B, blue), anti P-dAkt (A-B’, red) and anti-GFP (A, B, green) are shown. GFP expression (green) marks the expression domain of the apterous-Gal4 driver and the of the various expression constructs used. A’ and B’ show the P-dAkt channel only, the boundary of apterous-Gal4 expressing vs. non-expressing cells are marked with by a white line. Genotypes: (A, A’) yw; UAS-CD8::GFP, ap-Gal4/+; UAS-Tsc1, UAS-Tsc2/+. (B, B’) yw; UAS-CD8::GFP, ap-Gal4/UAS-S6KTE; UAS-Tsc1, UAS-Tsc2/+. (4.16 MB TIF) [file pgen.1000990.s007.tif]

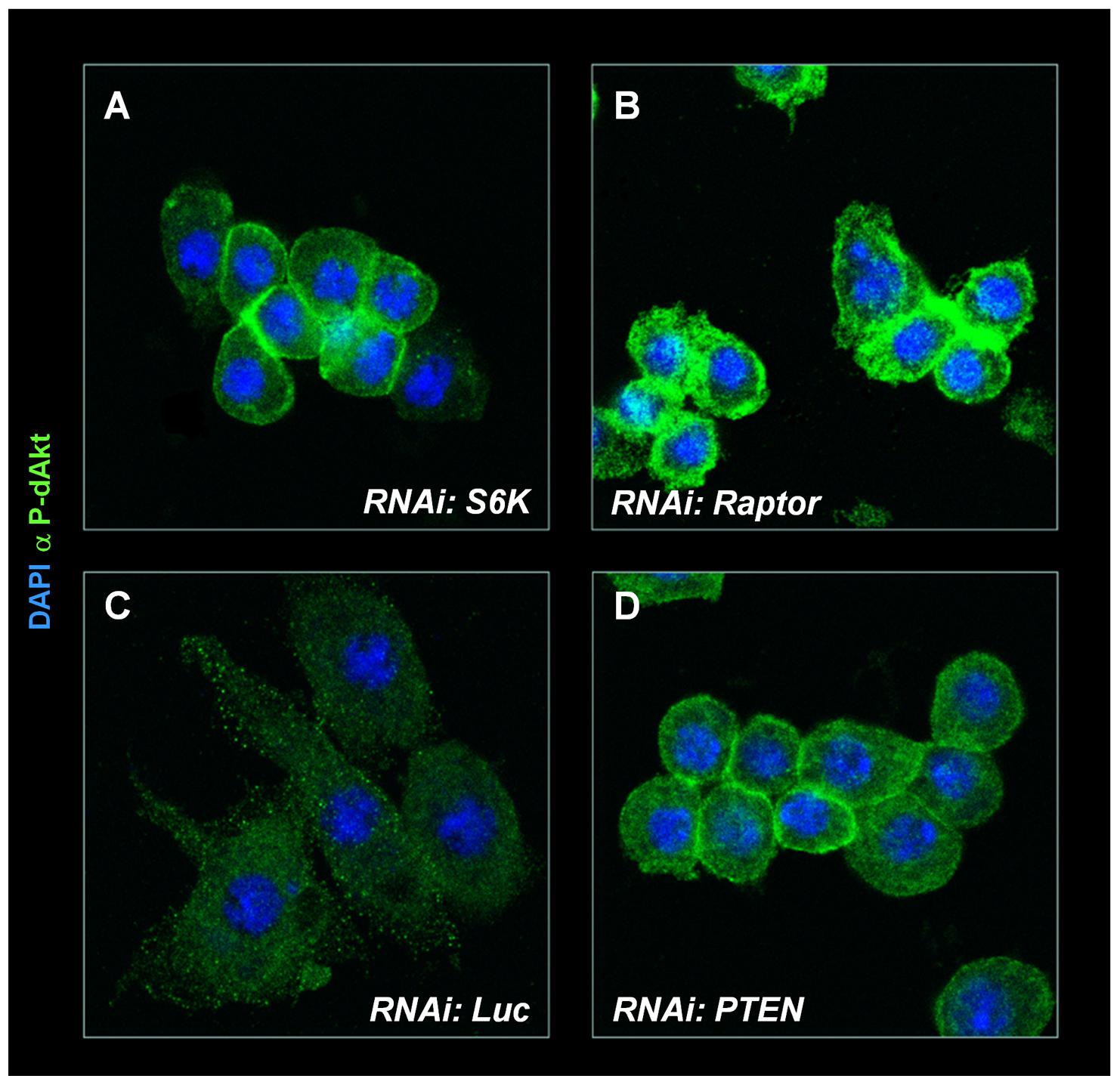

Supplement: Figure S8 — Raptor and S6K dependent negative feedback on P-dAkt. (A) Single confocal section of S6K, (B) Raptor, (C) Luciferase and (D) Pten RNAi treated Drosophila Kc167 cells stained with DAPI (blue) anti P-dAkt (green) after 10 minutes of insulin stimulation. Images were recorded and processed using identical conditions. Note the highest level of anti P-dAkt signal in the Raptor dsRNA treated cells. (2.64 MB TIF) [file pgen.1000990.s008.tif]
