# Supplementary material for: Dynamic Switch of Negative Feedback Regulation in Drosophila Akt–TOR Signaling
Source: PLoS Genet. 2010 Jun 17;6(6):e1000990. doi: 10.1371/journal.pgen.1000990 (PMC2887466; doi:10.1371/journal.pgen.1000990)
Supplement: Table S1 — Amplicons identified in the RNAi screens that enhance or suppress P-dAkt levels. Averaged Z-Scores from the two screen replicates of the baseline (no stimulation) and insulin-stimulated screens are shown. The DRSC amplicon identifies individual dsRNAs from the genome wide dsRNA set. Primer and sequence information available at www.flyRNAi.org. With the exception of the InR pathway components, the hits indicated in this Table were identified using a single dsRNA and therefore need further validation to eliminate false positives. Fbgn: Fly base gene number. (0.03 MB PDF) [file pgen.1000990.s009.pdf]

Gene Symbol	Gene name	Fbgn	DRSC Amplicon	Ave. Z-Score	Screen
Akt1	Akt1	FBgn0010379	DRSC14108	-9.8	Insulin Stimulated
rictor	rapa.-insensitive companion of Tor	FBgn0031006	DRSC20071	-8.8	Insulin Stimulated
sec13	sec13	FBgn0024509	DRSC14144	-7.1	Insulin Stimulated
InR	Insulin-like receptor	FBgn0013984	DRSC16717	-6.8	Insulin Stimulated
fliI	flightless I	FBgn0000709	DRSC20578	-6.1	Insulin Stimulated
AGO1	Argonaute-1	FBgn0026611	DRSC05912	-5.9	Insulin Stimulated
Sin1	SAPK-interacting protein 1	FBgn0033935	DRSC05965	-5.9	Insulin Stimulated
Nup133	CG6958	FBgn0039004	DRSC16150	-5.2	Insulin Stimulated
wdb	widerborst	FBgn0027492	DRSC14163	-5.0	Insulin Stimulated
CG7039	CG7039	FBgn0030088	DRSC18417	-4.7	Insulin Stimulated
Pi3K21B	Pi3K21B	FBgn0020622	DRSC00771	-4.5	Insulin Stimulated
Tsc1	Tsc1	FBgn0026317	DRSC16890	-4.4	Insulin Stimulated
Cpr49Ah	Cuticular protein 49Ah	FBgn0033731	DRSC07204	-4.4	Insulin Stimulated
CG3004	CG3004	FBgn0030142	DRSC18262	-4.3	Insulin Stimulated
CG33143	CG33143	FBgn0053143	DRSC04454	-4.2	Insulin Stimulated
CG10830	CG10830	FBgn0038839	DRSC14270	-3.9	Insulin Stimulated
CG32626	CG32626	FBgn0052626	DRSC19366	-3.8	Insulin Stimulated
Pink1	PTEN-induced putative kinase 1	FBgn0029891	DRSC18361	-3.8	Insulin Stimulated
l(1)10Bb	lethal (1) 10Bb	FBgn0001491	DRSC20346	-3.7	Insulin Stimulated
CG3756	CG3756	FBgn0031657	DRSC02719	-3.7	Insulin Stimulated
gig	gigas	FBgn0005198	DRSC11351	-3.5	Insulin Stimulated
SNF4Agamma	AMPK gamma subunit	FBgn0025803	DRSC16847	-3.4	Insulin Stimulated
eIF-1A	Eukaryotic initiation factor 1A	FBgn0026250	DRSC16937	-3.4	Insulin Stimulated
CG12267	CG12267	FBgn0038057	DRSC14490	-3.4	Insulin Stimulated
tinc	tincar	FBgn0038554	DRSC16309	-3.3	Insulin Stimulated
Hsc70-4	Heat shock protein cognate 4	FBgn0001219	DRSC16711	-3.2	Insulin Stimulated
CG9294	CG9294	FBgn0034666	DRSC04537	-3.2	Insulin Stimulated
eIF-2alpha	eIF-2alpha	FBgn0004925	DRSC20327	-3.2	Insulin Stimulated
CG5569	CG5569	FBgn0034919	DRSC04514	-3.2	Insulin Stimulated
CG4168	CG4168	FBgn0028888	DRSC01447	-3.1	Insulin Stimulated
CG11722	CG11722	FBgn0037777	DRSC14358	-3.1	Insulin Stimulated
CG5062	CG5062	FBgn0029747	DRSC18385	-3.1	Insulin Stimulated
Tak1	TGF-beta activated kinase 1	FBgn0026323	DRSC20390	-3.1	Insulin Stimulated
CG1115	CG1115	FBgn0037299	DRSC12165	-3.0	Insulin Stimulated
Dbp45A	DEAD box protein 45A	FBgn0010220	DRSC07395	-3.0	Insulin Stimulated
Hsc70-2	Heat shock protein cognate 2	FBgn0001217	DRSC16710	-2.9	Insulin Stimulated
sec23	sec23	FBgn0037357	DRSC12387	-2.8	Insulin Stimulated
drk	downstream of receptor kinase	FBgn0004638	DRSC07606	-2.8	Insulin Stimulated
Rpd3	Rpd3	FBgn0015805	DRSC08696	-2.8	Insulin Stimulated
CG11977	CG11977	FBgn0037650	DRSC14454	-2.8	Insulin Stimulated
eff	effete	FBgn0011217	DRSC16940	-2.8	Insulin Stimulated
DnaJ-1	DnaJ-like-1	FBgn0015657	DRSC11145	-2.8	Insulin Stimulated
CG5147	CG5147	FBgn0036775	DRSC10485	-2.8	Insulin Stimulated
eIF-4a	Eukaryotic initiation factor 4a	FBgn0001942	DRSC03526	-2.8	Insulin Stimulated
D19A	D19A	FBgn0022935	DRSC11133	-2.7	Insulin Stimulated
mts	microtubule star	FBgn0004177	DRSC03574	-2.7	Insulin Stimulated
CG11190	CG11190	FBgn0030035	DRSC17774	-2.7	Insulin Stimulated
CG32829	CG32829	FBgn0052829	DRSC00961	-2.7	Insulin Stimulated
Aats-arg	Arginyl-tRNA synthetase	FBgn0027093	DRSC20138	-2.7	Insulin Stimulated

Gene Symbol	Gene name	Fbgn	DRSC Amplicon	Ave. Z-Score	Screen
SNF1A	SNF1A/AMP-activated protein kinase	FBgn0023169	DRSC18714	-2.7	Insulin Stimulated
garz	gartenzwerg	FBgn0033714	DRSC07193	-2.7	Insulin Stimulated
foxo	forkhead box, sub-group O	FBgn0038197	DRSC15463	-2.7	Insulin Stimulated
Dhh1	DEAD/DEAH RNA helicase 1	FBgn0011802	DRSC11144	-2.7	Insulin Stimulated
CG31205	CG31205	FBgn0051205	DRSC13438	-2.6	Insulin Stimulated
blot	bloated tubules	FBgn0027660	DRSC11328	-2.6	Insulin Stimulated
CG7294	CG7294	FBgn0003209	DRSC03599	-2.6	Insulin Stimulated
CG31510	CG31510	FBgn0051510	DRSC14182	-2.6	Insulin Stimulated
CG12996	CG12996	FBgn0030835	DRSC19516	-2.6	Insulin Stimulated
Tor	Target of rapamycin	FBgn0021796	DRSC02811	-2.6	Insulin Stimulated
Srp72	Srp72	FBgn0038810	DRSC15800	-2.6	Insulin Stimulated
CG3701	CG3701	FBgn0034732	DRSC04409	-2.5	Insulin Stimulated
CG1358	CG1358	FBgn0033196	DRSC06405	-2.5	Insulin Stimulated
Lis-1	Lissencephaly-1	FBgn0015754	DRSC07459	-2.5	Insulin Stimulated
Notum	Notum	FBgn0044028	DRSC09959	-2.5	Insulin Stimulated
CG4415	CG4415	FBgn0031296	DRSC00654	-2.5	Insulin Stimulated
CG3332	CG3332	FBgn0031514	DRSC00594	-2.4	Insulin Stimulated
CG7546	CG7546	FBgn0035793	DRSC10871	-2.4	Insulin Stimulated
l(2)05070	lethal (2) 05070	FBgn0010590	DRSC07159	-2.4	Insulin Stimulated
CG32654	CG32654	FBgn0052654	DRSC19788	-2.4	Insulin Stimulated
zfh2	Zn finger homeodomain 2	FBgn0004607	DRSC17178	-2.4	Insulin Stimulated
crn	crooked neck	FBgn0000377	DRSC18755	-2.3	Insulin Stimulated
CG12090	CG12090	FBgn0035227	DRSC08249	-2.3	Insulin Stimulated
sktl	skittles	FBgn0016984	DRSC04715	-2.3	Insulin Stimulated
RpS5b	Ribosomal protein S5b	FBgn0038277	DRSC16173	-2.2	Insulin Stimulated
nuf	nuclear fallout	FBgn0013718	DRSC11379	-2.2	Insulin Stimulated
zetaCOP	zetaCOP	FBgn0040512	DRSC11412	-2.2	Insulin Stimulated
rdgB	retinal degeneration B	FBgn0003218	DRSC05596	-2.2	Insulin Stimulated
CG13217	CG13217	FBgn0033590	DRSC06339	-2.1	Insulin Stimulated
CG11136	CG11136	FBgn0034540	DRSC06045	2.1	Insulin Stimulated
CG9411	CG9411	FBgn0030569	DRSC20184	2.1	Insulin Stimulated
CG31158	CG31158	FBgn0051158	DRSC16141	2.1	Insulin Stimulated
Rheb	Rheb	FBgn0041191	DRSC12148	2.1	Insulin Stimulated
B4	B4	FBgn0023407	DRSC01868	2.1	Insulin Stimulated
CG13588	CG13588	FBgn0035038	DRSC04224	2.2	Insulin Stimulated
Ing3	Ing3	FBgn0030945	DRSC20002	2.2	Insulin Stimulated
CG15105	CG15105	FBgn0034412	DRSC06532	2.2	Insulin Stimulated
ham	hamlet	FBgn0045852	DRSC02070	2.3	Insulin Stimulated
CG9850	CG9850	FBgn0034903	DRSC04089	2.3	Insulin Stimulated
mip130	Myb interacting protein 130	FBgn0023509	DRSC18575	2.3	Insulin Stimulated
Rtnl1	Rtnl1	FBgn0053113	DRSC02684	2.3	Insulin Stimulated
CG9486	CG9486	FBgn0031791	DRSC03227	2.3	Insulin Stimulated
Pk61C	Protein kinase 61C	FBgn0020386	DRSC08682	2.5	Insulin Stimulated
CG8776	CG8776	FBgn0033764	DRSC07262	2.5	Insulin Stimulated
CG10301	CG10301	FBgn0039106	DRSC14226	2.6	Insulin Stimulated
loco	locomotion defects	FBgn0020278	DRSC16989	2.6	Insulin Stimulated
CG4068	CG4068	FBgn0029738	DRSC18342	2.6	Insulin Stimulated
Unc-89	Unc-89	FBgn0053519	DRSC04430	2.6	Insulin Stimulated
nrv1	nervana 1	FBgn0015776	DRSC03382	2.6	Insulin Stimulated

Gene Symbol	Gene name	Fbgn	DRSC Amplicon	Ave. Z-Score	Screen
CG14609	CG14609	FBgn0037483	DRSC12553	3.0	Insulin Stimulated
CG17255	CG17255	FBgn0030205	DRSC18123	3.5	Insulin Stimulated
sas	stranded at second	FBgn0002306	DRSC12631	3.6	Insulin Stimulated
CG34401	CG34401	FBgn0052542	DRSC19614	3.7	Insulin Stimulated
alt	aluminum tubes	FBgn0038535	DRSC15315	3.7	Insulin Stimulated
omd	oocyte maintenance defects	FBgn0038168	DRSC10347	3.0	Not Stimulated
CG18814	CG18814	FBgn0042137	DRSC10347	3.0	Not Stimulated
CG9723	CG9723	FBgn0030768	DRSC20206	3.0	Not Stimulated
CG14329	CG14329	FBgn0038525	DRSC14808	3.1	Not Stimulated
CG17472	CG17472	FBgn0032868	DRSC02622	3.1	Not Stimulated
CG32852	CG32852	FBgn0052852	DRSC12861	3.1	Not Stimulated
CG4877	CG4877	FBgn0036624	DRSC10454	3.1	Not Stimulated
Rheb	Rheb	FBgn0041191	DRSC12148	3.2	Not Stimulated
CG32103	CG32103	FBgn0052103	DRSC10415	3.2	Not Stimulated
Oseg6	Oseg6	FBgn0034452	DRSC06066	3.2	Not Stimulated
Pole2	Pole2	FBgn0035644	DRSC09737	3.2	Not Stimulated
Pde11	Phosphodiesterase 11	FBgn0085370	DRSC02028	3.2	Not Stimulated
Tapdelta	Translocon-associated protein delta	FBgn0021795	DRSC05946	3.2	Not Stimulated
CG14482	CG14482	FBgn0034245	DRSC06454	3.2	Not Stimulated
CG31272	CG31272	FBgn0051272	DRSC16058	3.3	Not Stimulated
CG3893	CG3893	FBgn0036826	DRSC10386	3.3	Not Stimulated
CG31525	CG31525	FBgn0051525	DRSC12004	3.3	Not Stimulated
PH4alphaEFB	prolyl-4-hydroxylase-alpha EFB	FBgn0039776	DRSC14950	3.4	Not Stimulated
CG12377	CG12377	FBgn0037168	DRSC11672	3.4	Not Stimulated
r-l	rudimentary-like	FBgn0003257	DRSC17042	3.4	Not Stimulated
CG10495	CG10495	FBgn0032750	DRSC02066	3.5	Not Stimulated
tacc	transforming acidic coiled-coil protein	FBgn0026620	DRSC12019	3.5	Not Stimulated
ari-2	ariadne 2	FBgn0025186	DRSC04672	3.5	Not Stimulated
S6k	RPS6-p70-protein kinase	FBgn0015806	DRSC11276	3.6	Not Stimulated
CG5171	CG5171	FBgn0031907	DRSC02823	3.6	Not Stimulated
CG13829	CG13829	FBgn0039059	DRSC14679	3.6	Not Stimulated
Adar	Adenosine deaminase acting on RNA	FBgn0026086	DRSC17725	4.1	Not Stimulated
CG17244	CG17244	FBgn0039031	DRSC15206	4.1	Not Stimulated
CG33310	CG33310	FBgn0053310	DRSC01897	4.4	Not Stimulated
CG4644	CG4644	FBgn0031300	DRSC00660	4.6	Not Stimulated
wge	winged eye	FBgn0051151	DRSC15672	4.7	Not Stimulated
Pk61C	Protein kinase 61C	FBgn0020386	DRSC08682	4.8	Not Stimulated
raptor	raptor	FBgn0029840	DRSC18359	7.5	Not Stimulated
